# A qualitative exploration of the low-resource tele-assisted home exercise program for balance and functional mobility in Parkinson’s disease (TELEPORT-PD)

**DOI:** 10.1371/journal.pdig.0001536

**Published:** 2026-06-30

**Authors:** Arnold Fredrick D’Souza, Tumkur Shivakumar Shwetha, Dushyanth Babu Jasti, Manikandan Natarajan

**Affiliations:** 1 Department of Physiotherapy, Manipal College of Health Professions, Manipal Academy of Higher Education, Manipal, India; 2 Symbiosis College of Physiotherapy, Symbiosis International (Deemed University), Pune, India; 3 Department of Clinical Psychology, Manipal College of Health Professions, Manipal Academy of Higher Education, Manipal, India; 4 Department of Neurology, Star Hospitals, Banjara Hills, Hyderabad, India; Polytechnic Institute of Porto: Instituto Politecnico do Porto, PORTUGAL

## Abstract

This study explores the perspectives of people with Parkinson’s disease (PD) on TELEPORT-PD, a low-resource tele-assisted home exercise program designed to improve balance and functional mobility. An exploratory qualitative study was conducted with eight participants who completed a two-week feasibility trial of TELEPORT-PD, followed by in-depth semi-structured interviews. Audio-recorded interviews were transcribed verbatim and analyzed using Microsoft Excel. Codes were assigned to themes derived from the Unified Theory of Acceptance and Use of Technology 2 (UTAUT-2) model. All participants reported positive experiences, with most encountering no major challenges. Many reported improvements in daily activities and valued physiotherapist-led exercise demonstrations, considering them more beneficial than conventional physiotherapy. A few suggested minor modifications. All participants expressed willingness to continue, and most would recommend the program to others with PD. TELEPORT-PD is well accepted, with most participants reporting functional improvements. The findings should be interpreted cautiously due to the small sample and potential response bias.

## Author summary

This study looked at the feedback of people with Parkinson’s disease about TELEPORT-PD, a newly developed low-cost remotely-supervised home exercise program to improve balance and mobility. Eight people were interviewed after having experienced the program for a two-week period. Their feedback was overwhelmingly positive. They highlighted improvements in their balance and mobility and found the program easy to perform. They appreciated the guidance provided by the physiotherapist via WhatsApp Messenger videoconferencing. They were satisfied with the program’s content and delivery, and expressed willingness to recommend it to others. In summary, TELEPORT-PD is a well-received and effective low-cost program for improving balance and mobility in persons with Parkinson’s disease over a two-week period.

## Introduction

Parkinson’s disease is the fastest-growing neurodegenerative condition worldwide [1]. This alarming rise in prevalence is of significant concern because of its looming impact on healthcare services. Notably, this pattern can also be observed in South Asia [2], where there is currently a dearth of research on Parkinson’s disease rehabilitation compared with that in the rest of the world.

Telerehabilitation saw a resurgence owing to its prominent role in enabling physiotherapy service delivery during the social distancing restrictions imposed during the COVID-19 pandemic. For persons with conditions that limit mobility, home-based telerehabilitation might allow them to participate in rehabilitation measures regardless of those restrictions [3]. Additionally, there are economic benefits, among others, that might make home-based telerehabilitation a suitable course of intervention [4–6].

The end recipients of an intervention must be active participants in the development of interventions for them. Their lived experience allows an informed application of the intervention, which takes into consideration all the unique requirements of the target population. This allows for the successful translation of clinical research into clinical practice [7,8]. Telerehabilitation for PD has been studied extensively in higher-income settings [9]; however, these experiences cannot be generalized for persons with PD outside that demographic. Differences in healthcare infrastructure, digital literacy, internet connectivity, socioeconomic status, access to technology, caregiver availability, and cultural perceptions of rehabilitation may substantially influence the feasibility, acceptability, and effectiveness of telerehabilitation programs. Consequently, interventions developed and evaluated in high-income settings may not adequately address the unique barriers and facilitators experienced by persons with PD living in lower-income or resource-constrained environments. Generating context-specific evidence is therefore essential to ensure that telerehabilitation models are relevant, accessible, and sustainable across diverse healthcare settings

Considering the requirements and limitations of low-resource settings, we developed a tele-assisted home exercise program for balance and functional mobility in persons with PD (TELEPORT-PD) via an international e-Delphi consensus and evaluated it in a feasibility study. Experiences of the trial participants provided unique insights into successfully deploying telerehabilitation in a context where participants had access to limited resources, underlining the need for more accessible healthcare delivery in such settings. The objective of this study was to understand and investigate the perspectives of participants toward the newly developed tele-assisted home exercise program for improving balance and functional mobility in persons with Parkinson’s disease. The qualitative approach was chosen to allow participants’ feedback to be studied and enhance TELEPORT-PD for use in a randomized controlled trial.

## Materials and methods

A qualitative study design was used, and in-depth, semi-structured interviews were conducted with each participant individually. The methodology and results are reported via the Consolidated Criteria for Reporting Qualitative Research (COREQ) checklist [10] (S2 File).

### Participants

The participants of this study were enrolled in the TELEPORT-PD feasibility trial [11]. This trial included adults with Parkinson’s disease diagnosed by a neurologist on the basis of the UK Parkinson’s Disease Society Brain Bank criteria. The participants were independent ambulators who did not require an assistive device, were staged between Hoehn and Yahr scales 1 and 3, and were on steady dopaminergic medication for two weeks before commencement. Additionally, participants were required to have an internet-enabled smart device (phone, tablet, or personal computer with WhatsApp installed). A functionally independent caregiver was required to be present during all the sessions to ensure safety and provide assistance. Individuals with significant cognitive impairment [Montreal Cognitive Assessment (MoCA) score under 21], significant fall risk (pull test score over 2), significant impairments in communication ability, coexisting medical conditions, or recent surgery that might affect their ability to safely participate in a structured exercise program, simultaneous involvement in any other exercise or physical activity program, and motor fluctuations and/or dyskinesias were excluded. The MoCA was used with permission for this study. All eight participants of the TELEPORT-PD feasibility trial were contacted via telephone and invited to participate in semi-structured interviews at a time and place of convenience.

### Ethics statement and informed consent

All participants provided written informed consent before inclusion. The study protocol was reviewed and approved by the Institutional Ethics Committee. The protocol was prospectively registered in the national clinical trials registry. Additionally, all the study participants were notified that this qualitative study was conducted as a part of the doctoral research work of the first author and that the research team had no ulterior motives or conflicts of interest.

### Intervention

A familiarization session was carried out in every participant’s home before commencement. The participants and caregivers were trained regarding the exercises, device, and environment setup, and troubleshooting. An exercise instruction manual and videos were provided. All participants received six sessions of one-on-one WhatsApp video call-assisted exercise training on non-consecutive days over two weeks. The physiotherapist provided a live exercise demonstration and guidance to the participants. The TELEPORT-PD protocol includes individually tailored multimodal exercises developed via e-Delphi consensus among a 15-member panel of experienced international Parkinson’s rehabilitation researchers and clinicians. The exercises are categorized into aerobic, resistance, balance, functional mobility, flexibility training, and relaxation techniques. Exercise intensity was set to moderate, as illustrated in Borg’s Rating of Perceived Exertion (RPE) scale.

### Interviewers and data coders

All the interviews were carried out by the first author, who is a third-year doctoral researcher with a Master’s degree in Physiotherapy and over five years of clinical experience in rehabilitation for persons with Parkinson’s disease. The second author is a Professor of Clinical Psychology with extensive qualitative research experience and provided the necessary guidance, training, and supervision to the first author, who conducted all the interviews. The fourth author is a Professor of Physiotherapy with extensive research experience and participated in coding the transcripts alongside the first author. In cases of disagreement between the coders, a consensus was reached after the second author was consulted.

### Assessments

The demographic details of all participants were collected during recruitment. All interviews were conducted the week following the end of the TELEPORT-PD feasibility trial. The first author conducted all the interviews in person. Five participants were interviewed in their homes, while three participants were interviewed in the physiotherapy outpatient department (OPD).

### Interviews and data collection

An in-depth interview guide was prepared. It was face and content validated by two experienced qualitative researchers who were not involved in this study. Both researchers had formal training in qualitative research and at least a decade of experience. The interview guide was pilot tested on two individuals who did not participate in the interviews. The pilot data were not included in the dataset. The suggested changes were incorporated, and the final interview guide was approved by both experts.

The interview guide included open-ended questions related to the participants’ experience with TELEPORT-PD, their likes, challenges faced, and modifications suggested, as well as the expected role of the physiotherapist and the caregiver. In addition, questions related to the comparison of telerehabilitation to in-person physiotherapy, as well as their willingness to continue the program, were included (S1 File).

Face-to-face interviews were conducted either at the participant’s home or in the physiotherapy outpatient department. All interviews were conducted in the presence of the caregiver. During the interviews, the participants were prompted to be candid and speak freely and at length about topics of particular significance to them. The duration of the interviews ranged from 30 to 45 minutes. All interviews were audio-recorded via a smartphone. Verbatim interview transcripts were prepared via Microsoft Word. Non-English interview transcripts were translated into English by a bilingual native speaker. Back translation was performed to ensure linguistic accuracy. The transcripts were sent to the participants to seek any additional input or modifications. There were no follow-up interviews. All potential identifiers were expunged from the transcripts before analysis.

### Data analysis

The interview transcripts were independently coded by two authors in Microsoft Excel, where a master spreadsheet was created with an audit trail log. A third author verified the codes and resolved any disagreements. The Unified Theory of Acceptance and Use of Technology 2 (UTAUT-2) model has seven key constructs that are used as themes to which the codes are assigned [12]. Various subthemes were created under each theme. The UTAUT-2 model has been utilized in the context of telerehabilitation acceptance among people with stroke [13]. Hence, it was chosen for use in this study due to similar rehabilitation challenges faced by persons with PD. Demographic characteristics were analysed via descriptive statistics in the EZR.

## Results

### Sample characteristics

Eight persons with PD participated in this qualitative study. The mean age of the participants was 67.5 years, with ages ranging from 58 to 76 years. Two participants were female, while the rest were male. The education status of the participants ranged from school degree to postgraduate degree, with half the participants having an undergraduate degree. The disease duration ranged from 0.5 to 13 years. The median disease severity was Hoehn and Yahr stage 2.5, while the median levodopa equivalent daily dose was 394.5 mg. The median MoCA score was 24 points. Five participants had their spouses as caregivers, while three participants had their adult children as caregivers. Except for one participant, all had received physiotherapy interventions at least three months before enrolment. All the participants received telerehabilitation for the first time. Half of the participants had some experience with the use of smartphone technology, but only one participant was accustomed to making videoconference calls over WhatsApp Messenger (Meta Platforms, Inc., California, USA) before enrolment. All the participants used smartphones for the sessions; six participants used mobile internet connections, while two had access to Wi-Fi at home. The average duration of individual sessions was 34 minutes. Table 1 describes the participants’ demographic characteristics.

**Table 1:** Participant characteristics.

ID	Age in years	Gender	Education	Occupation*	Location of residence	Caregiver	Time since diagnosis (Years)	LEDD (mg)	Hoehn and Yahr scale	MoCA score
P1	58	Female	Postgraduate	Pathologist	Suburban	Spouse	4	344	3	28
P2	72	Male	Postgraduate	Surgeon	Suburban	Spouse	7	606	2	25
P3	60	Male	High School	Bank manager	Rural	Son	2	424	2.5	22
P4	66	Male	Graduate	Clerk	Rural	Spouse	0.5	165	2	21
P5	76	Male	Graduate	Businessman	Suburban	Spouse	4	950	2.5	23
P6	65	Female	Graduate	Teacher	Suburban	Daughter	0.5	0	2.5	30
P7	70	Male	Graduate	Clerk	Rural	Son	13	2189	3	25
P8	73	Male	School	Agriculturist	Rural	Spouse	1	365	3	21

LEDD: Levodopa equivalent daily dosage.

MoCA: Montreal Cognitive Assessment.

*Except P1, all other participants were retired at the time of the interview.

### Qualitative findings

The UTAUT-2 model enabled the identification of seven themes: (1) performance expectancy, (2) effort expectancy, (3) social influence, (4) facilitating conditions, (5) hedonic motivation, (6) price value, and (7) habits. Table 2 shows all the themes and their underlying codes.

**Table 2:** Themes and codes derived from the qualitative data.

S No.	Themes	Codes
1	Performance expectancy	• Satisfaction with TELEPORT-PD • Improvement in health outcomes
2	Effort expectancy	• Technical challenges • Comparison to in-person rehabilitation
3	Social influence	• Active involvement of the physiotherapist • Presence of the caregiver
4	Facilitating conditions	• Simplicity • Lack of special equipment • Additional training resources
5	Hedonic motivation	• Positive interaction with the physiotherapist • Enjoyment
6	Price value	• Economic value compared with in-person rehabilitation
7	Habit	• Motivation to exercise • Willingness to continue • Customizability

1. **Performance expectancy**

**Satisfaction with TELEPORT-PD**

All the participants reported being satisfied with the program, whereas one notably remarked that their expectations were exceeded, highlighting the natural progression of the exercises and not realizing their rate of improvement. The participants also noted that the program was safe. The participants suggested that the session duration could be increased to 45 minutes.

P1 *“I would not say it met my expectation. I’d say it exceeded my expectations…I never realized that I was improving. It just was so naturally done. Therefore, I think this program actually exceeded my expectations.”*

P2 *“Yeah, the experience that I have had thus far, which is not a long duration, but whatever it is with telerehabilitation seems very positive. I personally liked it…I’m happy…I’m quite happy…I think it is quite safe.”*

P7 *“The timing is a little short I feel. I think 30 minutes is less. It should be at least 45 minutes long. I think.”*

**Improvements in health outcomes**

The participants specifically reported improvements in walking speed, strength, and flexibility. Additionally, the participants noted improvements in their confidence. They summarized their improvements as being both physical and mental.

P1 *“I used to have a lot of stiffness; axial stiffness and it was an impediment to normal daily activity - that disappeared…my walk became more fluid...I could keep up with my family when we went out together. Earlier, I was always trailing behind, now I can walk with them...”*

P5 *“Parkinson’s was slowly taking my strength... I met you at the right time; otherwise, I would have been depressed. (The)...strengthening exercises and mobility (exercises were) very good.”*

1. **Effort expectancy**

**Technical challenges**

Notably, the only challenge was the disruptions caused by internet connectivity. Sessions were continued without any problems after a brief delay. An in-person contact session at the beginning was highlighted to be necessary to allow the participant to be familiarized with the telerehabilitation setup.

P1 *“From the technical aspect, I think we had problems only once when there (were) some problems with the internet and we had to switch to WhatsApp video, but even then, we managed to complete the session without any problems.”*

P2 *“As I mentioned, as long as there is one contact session in the beginning, where you can show it. I think that is all that is necessary. The rest can easily be done in tele...”*

**Comparisons to in-person physiotherapy**

The comfort and privacy afforded at home were noted. The participants highlighted time savings and avoiding social interaction in addition to mobility restrictions that make commuting regularly to the physiotherapy outpatient department for sessions extremely difficult.

P1 *“…when you’re in an online program, you don’t have to deal with uh lots of people…waiting for the physiotherapist. Here you have dedicated time, and the physiotherapist is explaining things to you as we go along. So, your understanding of the process is a lot better. And I mean, considering the comfort and the privacy, I think that I consider the online program to be superior to regular physiotherapy. And now with…so many drug-resistant infections…no one really enjoys going to the hospital…It’s a lot safer. It’s very effective…I think it’s the way to go.”*

P8 *“Yeah, this online is better because coming to OPD, so many difficulties are there. Like one thing is crowd, then moving, getting out of the car, getting out of the vehicle, and then moving around. Waiting there. Those all problems we don’t face in online programs.”*

P6 *“Doing it at home is best for me…We have to wait for them…take appointments, and they will ask me to come at that time or this time…What I’m doing at home is best. No need of going out.”*

1. **Social influence**

**Active involvement of the physiotherapist**

The participants appreciated the demonstration of all the exercises in addition to the guidance provided by the physiotherapist throughout the session.

P1 *“...the demonstration of the exercises was very clear, making it easy to follow along. Although my camera was not always positioned to capture my feet or other relevant body parts, the physiotherapist, because of his experience, was still able to accurately assess my movements and provide precise feedback. There is nothing specific that I would suggest changing, as the current program meets all my expectations and needs.”*

P5 *“You are also describing very properly and very clearly, and there’s no difficulty…That’s very good. I like it.”*

**Supervision and assistance provided by the caregiver**

Since the program was not too challenging due to the graded evolution of the exercises, the participants did not feel the need for the caregiver to be more involved. The caregiver was not required to play an active role in the sessions and was mostly on standby to assist if needed and ensure the participant’s safety.

P1 *“It’s not a question of do you want or do you not want. It’s a question of where you are in the Parkinson’s journey and whether you need that extra help or whether you can manage on your own...I don’t really think that you need to have somebody sitting by your side all the time, but it’s my case.”*

1. **Facilitating conditions**

**Simplicity of TELEPORT-PD**

The program was conducted at home using technology that was already available. The participants found that the sessions were easy to carry out. Additionally, the exercises themselves were very easy to learn and perform.

P1 *“Though you’re using technology, it’s very simple - a communication between two people…having the program done in the privacy of your own home…with one-on-one interaction with the physiotherapist, dedicated time with them. I found lots of pluses in this online program.”*

P6 *“Really, it’s very good to me. These are the very good exercise. It’s enough for myself. I’m very active now. I like to do all these exercises. Easy to do…That is very nice. I can handle this easily.”*

**Lack of requirements for special equipment**

The exercises included in the program used easily available household objects for exercise delivery. There was no need for special equipment, which was appreciated by the participants.

P4 *“There is better equipment in the hospital…more equipment is used in clinics. At home, there is very little to no equipment. Exercises are similar — I cannot make out any difference when performing exercises at home.”*

**Additional training resources**

The participants were provided with an exercise instruction manual with illustrations and descriptions of all the included exercises with safety instructions. The participants were also provided with prerecorded videos of the exercise demonstration. These additional training resources were found to be detailed and self-explanatory. The categorization of exercises was noted to be of assistance. Some participants preferred watching the videos over reading the manual. The participants preferred a printed copy of the manual over a digital file.

P2 *“The illustrations and instructions are quite explicit…even in-person demonstration may not be necessary if people follow the instructions, because they seem very self-explanatory. I liked the categorization of exercises - strengthening, mobility and balance - once in separate categories, it becomes quite easy to follow on a regular basis.”*

P4 *“I would like a booklet of exercise in print to prevent distraction from the mobile (phone) while on the video call. To see the exercise since concentration is diverted on the mobile (phone). It is a problem.”*

P6 *“Exercise videos will be good to help in performing the exercise at home by ourselves…it is easier than with the booklet. I will be able to exercise with the videos and the pictures in the booklet. I am very happy overall.”*

1. **Hedonic motivation**

**Positive interaction with the physiotherapist**

The participants reported positive interaction with the physiotherapist, likening the interaction to being between a teacher and a student. They also appreciated patience, communication skills, and demonstrations provided by the therapist.

P3 stated, *“The session happens just the way it happens in a classroom, and it is easier to verify the doubts. The online session feels more like the happenings between a student and the teacher.”*

P8 *“Since it is a video call, it is more comfortable for us to do at home than to come to the hospital. Commutation is a problem. That is why we like your online session. Patience. Communication. Demonstration”*

**Enjoyment with TELEPORT-PD**

In contrast to their expectations, the participants felt that the program was interesting and fresh due to the progression of exercise without making them too difficult. They appreciated the natural progression of exercise, which allowed them to ease in and keep up with the program.

P1 *“I expected a very dull, dry do this do that sort of thing, which I’d have to force myself into. And it wasn’t like that at all. It was it was very interesting and. I mean, you kept it very fresh by introducing new things, and it was never difficult because I was sort of eased in slowly into the exercises. I never realized that I was improving. It just was so naturally done.”*

1. **Price value**

**Economic value compared with in-person physiotherapy**

Many participants reported saving money because they did not need to travel for their exercise sessions. Additionally, the relative savings in time and effort were appreciated as well.

P2 *“As long as there is one contact session in the beginning, I think that is all that is necessary. The rest can be easily performed via tele and it saves effort, time, money…I do not see any risk or difficulty, and I think showing once and then following up is a good idea. I should be able to do it with the video on my side for you to see the exercises I’m doing.”*

1. **Habit**

**Motivation to exercise**

The participants reported being motivated to exercise after participating in the program. They also described it as patient-friendly and appreciated its structure. This enhanced motivation for those who were not motivated at the beginning. Additionally, the participants reported that they are now more regular with exercise because of their introduction to the program.

P1 *“I would go to the extent of saying it was life changing because it managed to motivate me, a person who had zero motivation for exercise…I really benefited a lot from the program. It has been very well structured, very patient friendly, and it even motivates people who are not motivated to start off with. It’s a very effective program.”*

P2 *“I’m more involved in physio now than I have ever been…I was never very regular because getting into a routine is not easy - it’s not the exercises being difficult, it is the motivation. But since we started with the tele physio, I’ve been much more faithful…I would certainly recommend it to others in the same situation and would like some contact maybe once a month to know that things are going on.”*

**Customizability of TELEPORT-PD**

Since the program was developed specifically to address the requirements of a low-resource setting, it was readily applicable to the current patient group. The exercise sequence was noted to be adequately paced. Additionally, the flexibility of the number of sessions and their timing was appreciated.

P1 *“In India I just didn’t find anything that was doable or viable…there’s a big need for the development of online exercise programs in Parkinson’s because there must be lots of people like me who are looking for this option and it’s just not available at the present time.”*

P2 *“There’s no limitation of how many sessions I can do in the week because I’m doing it at home…it’s quite safe and adequately paced…upper and lower limb strengthening can be done comfortably in half an hour, which is a good amount of time for exercises.”*

**Willingness to continue the program**

The participants were willing to continue the exercise program because of its various benefits. They would also suggest that others with PD participate in the program. The participants appreciated the one-on-one sessions, which made interaction with the physiotherapist easier, leading to motivation and easier learning. Their opinion was not positive toward group telerehabilitation sessions, especially for individuals who are new to exercising via remote guidance.

P1 *“I would definitely recommend it to anybody without any hesitation…it was a great benefit to have a one-on-one interaction…you feel the involvement of another person and that motivates you, and things that need to be corrected are pointed out…I don’t know whether that’s possible in a big group. Maybe at a later stage it’s OK, but maybe not in the initial stages.”*

## Discussion

This qualitative study aimed to explore the perspectives of persons with Parkinson’s disease (PD) on the feasibility and acceptability of a low-resource tele-assisted home exercise program (TELEPORT-PD) designed to enhance balance and functional mobility. The application of the UTAUT-2 model facilitated an in-depth examination of key factors influencing the successful implementation of TELEPORT-PD in a feasibility trial. Unlike the original UTAUT and Technology Acceptance Model (TAM) which were developed for use by workplace employees, the UTAUT-2 was preferred due to the inclusion of Hedonic Motivation and Price Value, which are significant for persons using a technology-driven home-based intervention. Additionally, the UTAUT-2 covers a greater percentage of variance in behaviour intention in comparison to TAM. The findings of this study extend beyond simple descriptions of participant experience; rather, they reflect the underlying constructs of the UTAUT-2 framework that together shape behavioural intention, understood here as the likelihood that participants would continue engaging with TELEPORT-PD following the conclusion of the study. Across participants, several converging constructs pointed toward strong behavioural intention: perceived functional benefit (performance expectancy), ease of use (effort expectancy), enjoyment of the program (hedonic motivation), and perceived cost savings (price value). This convergence was evident in participants’ widespread willingness to continue with the program and to recommend it to others. The alignment of these multiple constructs suggests that TELEPORT-PD holds considerable promise for sustained real-world adoption. However, this potential remains contingent on ensuring the reliability of the underlying digital infrastructure and the continued availability of physiotherapist support, both of which emerged as important enabling conditions for long-term engagement.

The participants consistently expressed satisfaction despite their brief engagement in TELEPORT-PD, which was reinforced by perceived improvements in functional mobility, flexibility, and strength. This aligns with findings from previous qualitative studies in neurological populations, where participant satisfaction was closely linked to perceived functional benefits [14]. These insights underscore the importance of demonstrating clinical effectiveness during program development to enhance acceptance and sustained engagement. This could be achieved by objectively measuring participant satisfaction with telerehabilitation [15].

Technical challenges and comparisons with in-person physiotherapy shaped participants’ expectations regarding the effort required for engagement. Reliable internet connectivity has emerged as a crucial determinant of effective program delivery, highlighting the risk posed by inadequate digital infrastructure. Additionally, limited technological proficiency was identified as a barrier to participation. Deficiencies in appropriate technology and associated factors are known limitations of deploying a telerehabilitation intervention in a low-resource setting [16]. Providing structured training and involving caregivers in setup and troubleshooting are pivotal in mitigating these challenges. At the same time, home-based participation minimized travel-related burdens, conserving time and energy that could be redirected toward exercise performance, a critical consideration for individuals with PD experiencing mobility restrictions.

The active involvement of physiotherapists and the supportive presence of caregivers played a significant role in participant engagement. Compared with unsupervised exercise, supervised exercise programs have yielded better outcomes [17]. The physiotherapists’ clear instruction delivery and live demonstration of exercise fostered a strong therapeutic alliance, reinforcing motivation. Some participants noted that performing exercises alongside the physiotherapist resembled action observation therapy, potentially enhancing adherence. While participants did not perceive the caregiver as essential for exercise performance, their role in ensuring safety and providing reassurance was recognized as beneficial.

The simplicity of TELEPORT-PD, its reliance on readily available household items, and the provision of additional training resources facilitated ease of use. Familiarity with WhatsApp Messenger as a communication platform reduced the learning curve associated with the program. The exercises were designed to be simple and structured, with a focus on aerobic, resistance, flexibility, balance, and functional mobility training, as well as relaxation techniques. Supplementary materials, including printed and digital manuals with illustrations and pre-recorded videos, empowered participants to take an active role in self-management, potentially fostering long-term adherence.

Enjoyment and positive interactions with the physiotherapist emerged as key motivators. The participants described the physiotherapist’s role as akin to that of a teacher, emphasizing patience and effective communication. Effective communication by physiotherapists has been shown to improve a person’s experience with physiotherapy [18]. Initial concerns about the program being monotonous or overly demanding were alleviated by its structured progression, which maintained engagement while ensuring a gradual increase in exercise intensity.

Economic considerations were a significant factor influencing program acceptability. The cost-effectiveness of telerehabilitation, particularly in terms of reduced travel expenses and time savings, has been well documented [4–6]. The financial and logistical advantages of TELEPORT-PD may enhance long-term adherence, particularly for individuals with PD who require sustained rehabilitation. The impact of the program on long-term exercise adherence, however will need to be tested in a future randomized controlled trial with adequate follow-up period.

Motivation and program customizability are central to habit formation. The participants expressed a willingness to continue engaging in the program beyond the study period, reinforcing the potential for long-term adoption. Given the known challenges in sustaining exercise motivation among individuals with PD [19], the flexibility of TELEPORT-PD, including real-time feedback and exercise modifications on the basis of participant needs, appeared to foster a sense of empowerment. Safety considerations, such as adequate rest periods and support mechanisms, further reinforced participant confidence and program adherence.

The shorter duration of the program in contrast to other studies [9], still yielded a favourable motor outcome. In a similar vein, the perceived digital barriers reported in similar settings did not pose a significant barrier in participants adopting and accepting TELEPORT-PD. Furthermore, participants seem to suggest that the program could enable long-term adherence, even in persons who were not enthusiastic about the program outcomes in the beginning.

The overall positive response of most participants must be interpreted with caution. Several factors may have limited the objectivity of participant responses. Social desirability bias is particularly relevant here given that participants were interviewed by the clinician who delivered the intervention, a dynamic that may have discouraged candid criticism. Caregiver presence during interviews may have conferred a degree of emotional reassurance to participants, which could have consequentially influenced their responses. A novelty effect cannot be ruled out either, as initial enthusiasm for an unfamiliar modality may not reflect a durable appraisal of the program’s long-term value. Furthermore, cohort-specific characteristics, including high baseline motivation, prior physiotherapy experience, access to technology, and strong caregiver support, may have predisposed participants toward favourable perceptions. Collectively, these factors may account for the limited criticism expressed and caution against over-interpreting the findings as broadly representative. The experiences reported here may not generalize to individuals with limited technological access, less engaged caregivers, or lower initial motivation toward novel rehabilitation approaches.

Several measures were implemented to mitigate the potential influence of the interviewer’s dual role as their physiotherapist and interviewer. Prior to each interview, participants were explicitly encouraged to share their experiences candidly, including aspects they found difficult or disappointing, and were assured that their responses would bear no consequence on the care they received. To further reduce the potential influence of the interviewer’s preconceptions on the line of questioning, the interview guide was developed and validated by two independent experts in qualitative research who had no affiliation with the study. Finally, to introduce an additional layer of analytic distance, all transcripts were independently coded by a second author who had not been involved in the data collection process. Together, these strategies were intended to strengthen the credibility and reflexivity of the qualitative findings.

Overall, TELEPORT-PD addresses critical rehabilitation needs in specific resource-constrained settings. Future research should explore additional outcomes beyond balance and functional mobility to optimize the program’s impact.

### Strengths and limitations

This study provides novel insights into the perspectives of persons with PD regarding a low-resource telerehabilitation program, an area that has received limited attention. To the best of our knowledge, this is the first qualitative study in the region to explore this topic. The use of the UTAUT-2 model allowed for a comprehensive exploration of factors influencing program feasibility, whereas adherence to the COREQ checklist ensured rigor in data reporting.

However, certain limitations must be acknowledged. The small sample size limits the transferability and generalizability of the results. The participants’ perspectives were shaped by their first exposure to telerehabilitation within a brief two-week feasibility trial, which may have influenced their overwhelmingly positive responses. However, long-term acceptability and sustained engagement remain unexplored. Additionally, all participants had internet access, smart devices, and caregiver support, which may limit the generalizability of the findings to broader PD populations lacking these resources. Furthermore, both the therapy sessions and the subsequent interviews were conducted by the same investigator, which may have introduced bias in the participants’ responses. Given the feasibility constraints of the study, this limitation was unavoidable.

### Recommendations for future research

Future studies should focus on identifying and addressing the specific needs of persons with PD during the early development stages of telerehabilitation programs. Co-designing interventions with patients leads to superior utility and sustainable healthcare delivery. TELEPORT-PD can be scaled for deployment in other low-resource settings. Further research should evaluate long-term adherence, explore strategies to support participants with limited digital literacy or caregiver assistance, and investigate broader functional and psychosocial outcomes. An independent interviewer or data triangulation could be used in a future study to minimize potential bias.

## Conclusion

The participants expressed high levels of satisfaction with TELEPORT-PD, citing its accessibility, convenience, and adaptability. While initial technological barriers posed challenges, structured in-person familiarization facilitated a smooth transition to remote supervision via WhatsApp video calls. The program fostered motivation, engagement, and a sense of empowerment, with many participants expressing a desire for continued participation. These findings highlight the potential of low-resource telerehabilitation as a viable strategy for addressing rehabilitation needs in persons with PD, particularly in settings with limited access to in-person physiotherapy.

## Supporting information

S1 File Interview guide. (DOCX)

S2 File Checklist demonstrating that the manuscript conforms to the Consolidated criteria for reporting qualitative research (COREQ) Guidelines. (DOCX)
