# Supplementary material for: A qualitative exploration of the low-resource tele-assisted home exercise program for balance and functional mobility in Parkinson’s disease (TELEPORT-PD)
Source: PLOS Digit Health. 2026 Jun 30;5(6):e0001536. doi: 10.1371/journal.pdig.0001536 (PMC13318033; doi:10.1371/journal.pdig.0001536)
Supplement: S1 File — (DOCX) [file pdig.0001536.s001.docx]

**S1 File. Interview guide**

| **Questions** | **Probes** |
| --- | --- |
| How would you describe your experience of the home-based telerehabilitation program? | How does your experience compare to your expectations from the program? How did you feel during your participation in the program? Did you feel safe? |
| Please describe the challenges faced during the program. (Could you suggest modifications to improve the program?) | What were the challenges faced (if any)? How could we overcome these challenges? How did technology play a role in your experience with the program? How did you find the choice of technology used for the program? Have you had previous experience with such technology? Do you have suggestions for alternative choices of technology? |
| What are your expectations from the physiotherapist? | Should the physiotherapist do more to assist you? In what way would you prefer the physiotherapist assist you? |
| What are your expectations from the caregiver? | Which components did you like or dislike and why? What was missing from the program? What could have been omitted? How would you describe the difficulty level? Would you prefer to exercise in a group or individual setting? Please explain your choice. |
| How does this program compare to regular in-person physiotherapy? | Do you prefer telerehabilitation over regular physiotherapy? Why? What can we do to make telerehabilitation similar to regular physiotherapy |
| What do you expect from this program in the future if you continue it? | Would you prefer to continue home-based telerehabilitation in the future? What are your expectations in the long-term? |
| Do you have any additional remarks? | Would you like to add anything that we have not explored in this interview? |
