# Supplementary material for: A qualitative exploration of the low-resource tele-assisted home exercise program for balance and functional mobility in Parkinson’s disease (TELEPORT-PD)
Source: PLOS Digit Health. 2026 Jun 30;5(6):e0001536. doi: 10.1371/journal.pdig.0001536 (PMC13318033; doi:10.1371/journal.pdig.0001536)
Supplement: S2 File — (DOCX) [file pdig.0001536.s002.docx]

**S2 File. Checklist demonstrating that the manuscript conforms to the Consolidated criteria for reporting qualitative research (COREQ) Guidelines**

Domain 1: Research team and reflexivity

Personal Characteristics

1. Interviewer/facilitator

Which author/s conducted the interview or focus group?

- All interviews were conducted by the first author.

2. Credentials

What were the researcher’s credentials? E.g., PhD, MD

- The first author holds a Master of Physiotherapy (MPT) at the time of the study.

3. Occupation

What was their occupation at the time of the study?

- The first author was a third-year PhD student at the time of the study.

4. Gender

Was the researcher male or female?

- The first author is male.

5. Experience and training

What experience or training did the researcher have?

- The first author was trained and supervised by the second author, who is a professor of clinical psychology and has extensive experience in qualitative research.

Relationships with participants

6. Relationship established

Was a relationship established prior to study commencement?

- The participants participated in a two-week feasibility study before the interview.

7. Participant knowledge of the interviewer

What did the participants know about the researcher? e.g. personal goals, reasons for doing the research

- The participants were provided six sessions of video-guided home-based tele-rehabilitation over two weeks by the researcher. The participants were aware that the study was a part of the PhD work of the first author. They were informed of potential benefits such as improvements in balance and functional mobility. Additionally, the participants were informed that the results of this interview would be used to improve the program.

8. Interviewer characteristics

What characteristics were reported about the interviewer/facilitator? e.g. Bias, assumptions, reasons, and interests in the research topic.

- The various characteristics of the researchers are reported in the manuscript.

Domain 2: study design

Theoretical framework

9. Methodological orientation and theory

What methodological orientation was stated to underpin the study? e.g., grounded theory, discourse analysis, ethnography, phenomenology, content analysis

- Content analysis was used to analyze the qualitative data.

Participant selection

10. Sampling

How were participants selected? e.g. purposive, convenience, consecutive, snowball

- All participants underwent a 2-week feasibility trial. They were selected via a purposive sampling method.

11. Method of approach

How were participants approached? e.g., face-to-face, telephone, mail, email

- The participants were identified by screening patients who were visiting the Neurology OPD of the hospital.

12. Sample size

How many participants were in the study?

- There were eight participants in the study.

13. Non participation

How many people refused to participate or dropped out? Reasons?

- None.

Setting

14. Setting of data collection

Where was the data collected? e.g., home, clinic, workplace

- Five participants were interviewed at their respective homes. Three participants were interviewed at the clinic.

15. Presence of non-participants

Was anyone else present besides the participants and researchers?

- Six participants were accompanied by their spouses. Two participants had their adult children accompany them.

16. Description of the sample

What are the important characteristics of the sample? e.g. demographic data, date

- The demographic information of the sample is provided in Table 1.

Data collection

17. Interview guide

Were questions, prompts, and guides provided by the authors? Was it pilot tested?

- The questions, prompts, and guides were provided by the authors. They were validated by consultation with two experienced qualitative researchers and pilot tested with non-participants.

18. Repeat interviews

Were repeat interviews carried out? If yes, how many?

- No.

19. Audio/visual recording

Did the research use audio or visual recording to collect the data?

- The research used audio recordings for data collection.

20. Field notes

Were field notes made during and/or after the interview or focus group?

- No.

21. Duration

What was the duration of the interviews or focus group?

- Each interview took approximately 30 to 40 minutes.

22. Data saturation

Was data saturation discussed?

- We coded the data until saturation was reached.

23. Transcripts returned

Were transcripts returned to participants for comment and/or correction?

- Yes.

Domain 3: analysis and findings

Data analysis

24. Number of data coders

How many data coders coded the data?

- Two coders coded the data: the first and second authors.

25. Description of the coding tree

Did the authors provide a description of the coding tree?

- Yes.

26. Derivation of themes

Were the themes identified in advance or derived from the data?

- The themes were developed by following the Unified Theory of Acceptance and Use of Technology (UTAUT) model, and the subthemes were subsequently derived from the data.

27. Software

What software, if applicable, was used to manage the data?

- Microsoft Excel was used to manage the data.

28. Participant checking

Did participants provide feedback on the findings?

- No.

Reporting

29. Quotations presented

Were participant quotations presented to illustrate the themes/findings? Was each quotation identified? e.g. participant number

- Yes.

30. Data and findings consistent

Was there consistency between the data presented and the findings?

- Yes.

31. Clarity of major themes

Were major themes clearly presented in the findings?

- Yes.

32. Clarity of minor themes

Is there a description of diverse cases or a discussion of minor themes?

- Yes.
