# Supplementary material for: A qualitative exploration of the low-resource tele-assisted home exercise program for balance and functional mobility in Parkinson’s disease (TELEPORT-PD)
Source: PLOS Digit Health. 2026 Jun 30;5(6):e0001536. doi: 10.1371/journal.pdig.0001536 (PMC13318033; doi:10.1371/journal.pdig.0001536)
Supplement: Attachment — Submitted filename: PDIG-D-25-00578.pdf [file pdig.0001536.s003.pdf]

PLOS Digital Health

Exploring participants' perspectives toward a low-resource tele-assisted home exercise program for balance and functional mobility in Parkinson's disease (TELEPORT-PD): a qualitative study from India

--Manuscript Draft--

**Manuscript Number:**	PDIG-D-25-00578
**Article Type:**	Research Article
**Full Title:**	Exploring participants' perspectives toward a low-resource tele-assisted home exercise program for balance and functional mobility in Parkinson's disease (TELEPORT-PD): a qualitative study from India
**Short Title:**	Participants' experiences of the low-resource TELEPORT-PD program
**Corresponding Author:**	Manikandan Natarajan<br>Manipal College of Health Professions<br>Manipal, Karnataka INDIA
**Order of Authors:**	Arnold Fredrick D'Souza
	Shwetha T.S.
	Dushyanth Babu Jasti
	Manikandan Natarajan
**Keywords:**	Parkinson disease; Telehealth; Balance; Mobility; Qualitative Study
**Abstract:**	This study explores the perspectives of people with Parkinson's disease (PD) on TELEPORT-PD, a low-resource tele-assisted home exercise program designed to improve balance and functional mobility. A qualitative study was conducted with eight participants who completed a two-week feasibility trial of TELEPORT-PD, followed by in-depth semi-structured interviews. Audio-recorded interviews were transcribed verbatim and analyzed using Microsoft Excel. Codes were assigned to themes derived from the Unified Theory of Acceptance and Use of the Technology 2 (UTAUT-2) model. All participants reported positive experiences, with most encountering no major challenges. Many observed improvements in daily activities and valued physiotherapist-led exercise demonstrations, considering them more beneficial than conventional physiotherapy. A few suggested minor modifications. All participants expressed willingness to continue, and most would recommend the program to others with PD. TELEPORT-PD is well accepted, with most participants reporting functional improvements.
**Additional Information:**	
**Question**	**Response**
**Government Employee**<br><br>Are you or any of the contributing authors an employee of the United States government?<br><br>Manuscripts authored by one or more US Government employees are not copyrighted, but are licensed under a CC0 Public Domain Dedication, which allows unlimited distribution and reuse of the article for any lawful purpose. This is a legal requirement for US Government	No - No authors are employees of the U.S. government.

Powered by Editorial Manager® and ProduXion Manager® from Aries Systems Corporation

employees.<br><br>This will be typeset if the manuscript is accepted for publication.	
**Financial Disclosure**<br><br>Enter a financial disclosure statement that describes the sources of funding for the work included in this submission. Review the submission guidelines for detailed requirements. View published research articles from *PLOS Digital Health* for specific examples.<br><br>This statement is required for submission and **will appear in the published article** if the submission is accepted. Please make sure it is accurate.<br><br>**Funded studies**<br>Enter a statement with the following details:<br>• Initials of the authors who received each award<br>• Grant numbers awarded to each author<br>• The full name of each funder<br>• URL of each funder website<br>• Did the sponsors or funders play any role in the study design, data collection and analysis, decision to publish, or preparation of the manuscript?<br><br>Did you receive funding for this work?	The author(s) received no specific funding for this work.
**Competing Interests**<br><br>On behalf of all authors, disclose any competing interests that could be perceived to bias this work.<br><br>This statement will be typeset if the manuscript is accepted for publication.<br><br>Review the instructions link below and PLOS Digital Health's competing interests policy to determine what information must	The authors have declared that no competing interests exist.

be disclosed at submission.	
**Data Availability**<br><br>Provide a **Data Availability Statement** in the box below. This statement should detail where the data used in this submission can be accessed. This statement will be typeset if the manuscript is accepted for publication.<br><br>Before publication, authors are required to make all data underlying their findings fully available, without restriction. Review our PLOS Data Policy page for detailed information on this policy. Instructions for writing your Data Availability statement can be accessed via the Instructions link below.	All relevant data are within the manuscript and its Supporting Information files.

Manuscript
Click here to access/download;Manuscript;PLoS Digital Health Manuscript - Without Field Codes.docx

Full Title

Exploring participants' perspectives toward a low-resource tele-assisted home exercise program for balance and functional mobility in Parkinson's disease (TELEPORT-PD): a qualitative study from India

Short Title

Participants' experiences of the low-resource TELEPORT-PD program

Authors

Arnold Fredrick D'Souza[1], Shwetha T.S.[2], Dushyanth Babu Jasti[3], Manikandan Natarajan[1*]

Affiliations

[1]Department of Physiotherapy, Manipal College of Health Professions, Manipal Academy of Higher Education, Manipal, Karnataka, India

[2]Department of Clinical Psychology, Manipal College of Health Professions, Manipal Academy of Higher Education, Manipal, Karnataka, India

[3]Department of Neurology, Star Hospitals, Banjara Hills, Hyderabad, Telangana, India

*Corresponding author

E-mail: mani.kandan@manipal.edu

Author contributions

AFD: Conceptualization, Formal analysis, Investigation, Methodology, Writing – original draft; **STS:** Data curation, Formal analysis, Methodology, Validation, Writing – review &

editing; **DBJ:** Conceptualization, Methodology, Project administration, Supervision, Writing – review & editing; **MN:** Conceptualization, Data curation, Formal analysis, Methodology, Project administration, Supervision, Validation, Visualization, Writing – review & editing

Abstract

This study explores the perspectives of people with Parkinson's disease (PD) on TELEPORT-PD, a low-resource tele-assisted home exercise program designed to improve balance and functional mobility. A qualitative study was conducted with eight participants who completed a two-week feasibility trial of TELEPORT-PD, followed by in-depth semi-structured interviews. Audio-recorded interviews were transcribed verbatim and analyzed using Microsoft Excel. Codes were assigned to themes derived from the Unified Theory of Acceptance and Use of the Technology 2 (UTAUT-2) model. All participants reported positive experiences, with most encountering no major challenges. Many observed improvements in daily activities and valued physiotherapist-led exercise demonstrations, considering them more beneficial than conventional physiotherapy. A few suggested minor modifications. All participants expressed willingness to continue, and most would recommend the program to others with PD. TELEPORT-PD is well accepted, with most participants reporting functional improvements.

Keywords: Parkinson disease, Telehealth, Balance, Mobility, Qualitative study

Introduction

Parkinson’s disease is the fastest-growing neurodegenerative condition worldwide [1]. This alarming rise in prevalence is of significant concern because of its looming impact on healthcare services. Notably, this pattern can also be observed in South Asia [2], where there is currently a dearth of research on Parkinson's disease rehabilitation compared with that in the rest of the world.

Telerehabilitation has re-emerged as a rehabilitation tool because of its prominent role in enabling physiotherapy service delivery during the social distancing restrictions imposed during the COVID-19 pandemic. For persons with conditions that limit mobility, home-based telerehabilitation might allow them to participate in rehabilitation measures regardless of those restrictions [3]. Additionally, there are economic benefits, among others, that might make home-based telerehabilitation a suitable course of intervention [4–6].

The end recipients of an intervention must be active participants in the development of interventions for them. Their lived experience allows an informed application of the intervention, which takes into consideration all the unique requirements of the target population. This allows for the successful translation of clinical research into clinical practice [7,8]. Considering the requirements and limitations of low-resource settings, we developed a tele-assisted home exercise program for balance and functional mobility in PD patients (TELEPORT-PD) via an international e-Delphi consensus and tested it in a feasibility study. The objective of this study was to understand and investigate the perspectives of participants toward the newly developed tele-assisted home exercise program for improving balance and functional mobility in persons with Parkinson’s disease. The qualitative approach was chosen to allow participants’ feedback to be studied and enhance TELEPORT-PD for use in a randomized controlled trial.

Materials and methods

A qualitative study design was used, and in depth, semi structured interviews were conducted individually. The methodology and results are reported via the Consolidated Criteria for Reporting Qualitative Research (COREQ) checklist [9].

Participants

The participants of this study were enrolled in the TELEPORT-PD feasibility trial. This trial included adults with Parkinson's disease diagnosed by a neurologist on the basis of the UK Parkinson's Disease Society Brain Bank criteria. The participants were independent ambulators who did not require an assistive device, were staged between Hoehn and Yahr scales 1 and 3, and were on steady dopaminergic medication for two weeks before commencement. Additionally, participants were required to have an internet-enabled smart device (phone, tablet, or personal computer with WhatsApp installed). A functionally independent caregiver was required to be present during all the sessions to ensure safety and provide assistance. Individuals with significant cognitive impairment [Montreal Cognitive Assessment (MoCA) score under 21], significant fall risk (pull test score over 2), significant impairments in communication ability, coexisting medical conditions, or recent surgery that might affect their ability to safely participate in a structured exercise program, simultaneous involvement in any other exercise or physical activity program, and motor fluctuations and/or dyskinesias were excluded. The MoCA was used with permission for this study. All eight participants of the TELEPORT-PD feasibility trial were contacted via telephone and invited to participate in semi structured interviews at a time and place of convenience.

Ethics statement and informed consent

All participants provided written informed consent before inclusion. The study protocol was reviewed and approved by the Institutional Ethics Committee. The protocol was

prospectively registered in the national clinical trials registry. Additionally, all the study participants were notified that this qualitative study was conducted as a part of the doctoral research work of the first author and that the research team had no ulterior motives or conflicts of interest.

Intervention

A familiarization session was carried out in every participant's home before commencement. The participants and caregivers were trained regarding the exercises, device, and environment setup, and troubleshooting. An exercise instruction manual and videos were provided. All participants received six sessions of one-on-one WhatsApp video call-assisted exercise training on non-consecutive days over two weeks. The physiotherapist provided a live exercise demonstration and guidance to the participants. The TELEPORT-PD protocol includes individually tailored multimodal exercises developed via e-Delphi consensus among a 15-member panel of experienced international Parkinson's rehabilitation researchers and clinicians. The exercises are categorized into aerobic, resistance, balance, functional mobility, flexibility training, and relaxation techniques. Exercise intensity was set to moderate, as illustrated in Borg's Rating of Perceived Exertion (RPE) scale.

Interviewers and data coders

All the interviews were carried out by the first author, who is a third-year doctoral researcher with a Master's degree in Physiotherapy and over five years of clinical experience in rehabilitation for persons with Parkinson's disease. The second author is a Professor of Clinical Psychology with extensive qualitative research experience and provided the necessary guidance, training, and supervision to the first author, who conducted all the interviews. The fourth author is a Professor of Physiotherapy with extensive research experience and participated in coding the transcripts alongside the first author. In cases of

disagreement between the coders, a consensus was reached after the second author was consulted.

Assessments

The demographic details of all participants were collected during recruitment. All interviews were conducted the week following the end of the TELEPORT-PD feasibility trial. The first author conducted all the interviews in person. Five participants were interviewed in their homes, while three participants were interviewed in the physiotherapy outpatient department (OPD).

Interviews and Data collection

An in-depth interview guide was prepared. It was validated by two experienced qualitative researchers who were not involved in this study. The interview guide was pilot tested on two individuals who did not participate in the interviews. The suggested changes were incorporated, and the final interview guide was approved by both experts.

The interview guide included open-ended questions related to the participants’ experience with TELEPORT-PD, their likes, challenges faced, and modifications suggested, as well as the expected role of the physiotherapist and the caregiver. In addition, questions related to the comparison of telerehabilitation to in-person physiotherapy, as well as their willingness to continue the program, were included.

Face-to-face interviews were conducted either at the participant’s home or in the physiotherapy outpatient department. All interviews were conducted in the presence of the caregiver. During the interviews, the participants were prompted to be candid and speak freely and at length about topics of particular significance to them. The duration of the interviews ranged from 30 to 45 minutes. All interviews were audio-recorded via a smartphone. Verbatim interview transcripts were prepared via Microsoft Word. Non-English

interview transcripts were translated into English by a bilingual native speaker. The transcripts were sent to the participants to seek any additional input or modifications. There were no follow-up interviews. All potential identifiers were expunged from the transcripts before analysis.

Data analysis

The interview transcripts were independently coded by two authors in Microsoft Excel. A third author verified the codes and resolved any disagreements. The Unified Theory of Acceptance and Use of Technology 2 (UTAUT-2) model has seven key constructs that are used as themes to which the codes are assigned [10]. Various subthemes were created under each theme. The UTAUT-2 model has been utilized in the context of telerehabilitation acceptance among people with stroke [11]. Demographic characteristics were analysed via descriptive statistics in the EZR.

Results

Sample characteristics

Eight persons with PD participated in this qualitative study. The mean age of the participants was 67.5 years, with ages ranging from 58 to 76 years. Two participants were female, while the rest were male. The education status of the participants ranged from school degree to postgraduate degree, with half the participants having an undergraduate degree. The disease duration ranged from 0.5 to 13 years. The median disease severity was Hoehn and Yahr stage 2.5, while the median levodopa equivalent daily dose was 394.5 mg. The median MoCA score was 24 points. Five participants had their spouses as caregivers, while three participants had their adult children as caregivers. Except for one participant, all had received

physiotherapy interventions at least three months before enrolment. All the participants received telerehabilitation for the first time. Half of the participants had some experience with the use of smartphone technology, but only one participant was accustomed to making videoconference calls over WhatsApp Messenger (Meta Platforms, Inc., California, USA) before enrolment. All the participants used smartphones for the sessions; six participants used mobile internet connections, while two had access to Wi-Fi at home. The average duration of individual sessions was 34 minutes. Table 1 describes the participants' demographic characteristics.

Table 1. Participant characteristics

**ID**	**Age in years**	**Gender**	**Education**	**Occupation***	**Location of residence**	**Caregiver**	**Time since diagnosis (Years)**	**LEDD (mg)**	**Hoehn and Yahr scale**	**MoCA score**
P1	58	Female	Postgraduate	Pathologist	Suburban	Spouse	4	344	3	28
P2	72	Male	Postgraduate	Surgeon	Suburban	Spouse	7	606	2	25
P3	60	Male	High School	Bank manager	Rural	Son	2	424	2.5	22
P4	66	Male	Graduate	Clerk	Rural	Spouse	0.5	165	2	21
P5	76	Male	Graduate	Businessman	Suburban	Spouse	4	950	2.5	23
P6	65	Female	Graduate	Teacher	Suburban	Daughter	0.5	0	2.5	30
P7	70	Male	Graduate	Clerk	Rural	Son	13	2189	3	25
P8	73	Male	School	Agriculturist	Rural	Spouse	1	365	3	21

LEDD: Levodopa equivalent daily dosage

MoCA: Montreal Cognitive Assessment

*Except P1, all other participants were retired at the time of the interview.

Qualitative findings

The UTAUT-2 model enabled the identification of seven themes: (1) performance expectancy, (2) effort expectancy, (3) social influence, (4) facilitating conditions, (5) hedonic motivation, (6) price value, and (7) habits. Table 2 shows all the themes and their underlying codes.

Table 2. Themes and codes derived from the qualitative data

S No	Themes	Codes
1	Performance expectancy	• Satisfaction with TELEPORT-PD<br>• Improvement in health outcomes
2	Effort expectancy	• Technical challenges<br>• Comparison to in-person rehabilitation
3	Social influence	• Active involvement of the physiotherapist<br>• Presence of the caregiver
4	Facilitating conditions	• Simplicity<br>• Lack of special equipment<br>• Additional training resources
5	Hedonic motivation	• Positive interaction with the physiotherapist<br>• Enjoyment
6	Price value	• Economic value compared with in-person rehabilitation
7	Habit	• Motivation to exercise<br>• Willingness to continue<br>• Customizability

1. Performance expectancy

Satisfaction with TELEPORT-PD

All the participants reported being satisfied with the program, whereas one notably remarked that their expectations were exceeded, highlighting the natural progression of the exercises

and not realizing their rate of improvement. The participants also noted that the program was safe. The participants suggested that the session duration could be increased to 45 minutes.

P1 *"I would not say it met my expectation. I'd say it exceeded my expectations…I never realized that I was improving. It just was so naturally done. Therefore, I think this program actually exceeded my expectations."*

P2 *"Yeah, the experience that I have had thus far, which is not a long duration, but whatever it is with telerehabilitation seems very positive. I personally liked it…I'm happy…I'm quite happy…I think it is quite safe."*

P7 *"The timing is a little short I feel. I think 30 minutes is less. It should be at least 45 minutes long. I think."*

Improvements in health outcomes

The participants specifically reported improvements in walking speed, strength, and flexibility. Additionally, the participants noted improvements in their confidence. They summarized their improvements as being both physical and mental.

P1 *"I used to have a lot of stiffness; axial stiffness and it was an impediment to normal daily activity - that disappeared. I was really slow when I walked...but I feel that with the program, my walk became more fluid... I could keep up with my family when we went out together. Earlier, I was always trailing behind, now I can walk with them... You become more confident. So…not only physical(ly), but even mentally, I have benefited from the program."*

P5 *"Parkinson's was slowly taking my strength... I met you at the right time; otherwise, I would have been depressed. (The)...strengthening exercises and mobility (exercises were) very good."*

2. Effort expectancy

Technical challenges

Notably, the only challenge was the disruptions caused by internet connectivity. Sessions were continued without any problems after a brief delay. An in-person contact session at the beginning was highlighted to be necessary to allow the participant to be familiarized with the telerehabilitation setup.

P1 *"From the technical aspect, I think we had problems only once when there (were) some problems with the internet and we had to switch to WhatsApp video, but even then, we managed to complete the session without any problems, and I don't think the way it was done for me. I don't really see any serious (problem)."*

P2 *"As I mentioned, as long as there is one contact session in the beginning. Where you can show it. I think that is all that is necessary. The rest can easily be done in tele... What I would suggest is maybe at some point where you want to check I should be able to do it with the video on my side for you to see what the exercises that I'm doing when I'm doing it OK. So that can be done after a few weeks or whatever."*

Comparisons to in-person physiotherapy

The comfort and privacy afforded at home were noted. The participants highlighted time savings and avoiding social interaction in addition to mobility restrictions that make commuting regularly to the physiotherapy outpatient department for sessions extremely difficult.

P1 *"I think you know the thing about when you compare the two, when you're in an online program, you don't have to deal with uh lots of people, crowds waiting for the physiotherapist. Here you have dedicated time, and the physiotherapist is explaining things to you as we go along. So, your understanding of the process is a lot better. And I mean, considering the comfort and the privacy, I think that I consider the online program to be superior to the regular physiotherapy. And now with, you know, so many drug-resistant infections coming around no one really enjoys going to the hospital and hanging out unless you absolutely have to. So, I think online is the way to go. It's a lot safer. It's very effective. So why not? I think it's the way to go."*

P8 *"Yeah, this online is better because coming to OPD, so many difficulties are there. Like one thing is crowd, then moving, getting out of the car, getting out of the vehicle, then moving around. Waiting there. Those all problems we don't face in online programs."*

P6 *"Doing it at home is best for me. Not for clinic, clinic. We have to wait for them and take appointments, and they will ask me to come at that time or this time; it's comfortable. What I'm doing at home is best. No need of going out."*

3. Social influence

Active involvement of the physiotherapist

The participants appreciated the demonstration of all the exercises in addition to the guidance provided by the physiotherapist throughout the session.

P1 *"...the demonstration of the exercises was very clear, making it easy to follow along. Although my camera was not always positioned to capture my feet or other relevant body parts, the physiotherapist, because of his experience, was still able to accurately assess my movements and provide precise feedback. There is nothing specific that I would suggest changing, as the current program meets all my expectations and needs."*

P5 *"You are also describing very properly and very clearly, and there's no difficulty in taking this one. That's very good. I like it."*

P6 *"...because of your explanation and you made everything that is really I like very much. Oh my. Because. So, it was very nice. Nicely. You explained. You taught me. Really. It's very, very happy. I'm very glad to you. Thank you so much."*

Supervision and assistance provided by the caregiver

Since the program was not too challenging due to the graded evolution of the exercises, the participants did not feel the need for the caregiver to be more involved. The caregiver was not required to play an active role in the sessions and was mostly on standby to assist if needed and ensure the participant's safety.

P1 *"I think that would depend upon where you are in your Parkinson's journey. I'm fortunately, at a place where I'm reasonably independent. I don't need anyone to help me around. So, it's been OK to do. To do most of this without a caregiver. Actually, you know helping me, but it's possible that later down the road I may need the help of a caregiver. So, I think it really depends. It's not it's not a question of do you want or do you not want. It's a question of where you are in the Parkinson's....journey and whether you need that extra help or whether you can manage on your own. I think the exercises were done in such a careful graded manner that there were no risky points. I didn't feel at any point that I was put to any risk and for that reason I didn't feel the necessity of having a caregiver...Sitting right by my side. In fact, the initial sessions on balance, my caregiver was always sitting at hands distant. But that actually made me feel guilty because that person was tied down for that much time without really having anything to do. So, I felt oh I'm, you know, putting somebody through a lot of trouble. So, I think the way the exercises have been introduced and the way they're graded and. I think you really kept things safe. I mean very often you said safety first, so. I don't really think that you need to have somebody sitting by your side all the time, but it's my case."*

4. Facilitating conditions

Simplicity of TELEPORT-PD

The program was conducted at home using technology that was already available. The participants found that the sessions were easy to carry out. Additionally, the exercises themselves were very easy to learn and perform.

P1 *"Well, one thing I liked was the simplicity of the whole thing. It's, I mean, though you're using technology, but the actual ground thing is very simple. A communication between two*

people. And there's the added advantage of having the program done in the privacy of your own home. You don't have to go out and adjust to being in front of a whole number of people, which sort of puts me off and doing the program in the privacy of your own home and without having to go out and get dressed to do it and wait to connect with the physiotherapist. You have a really effective program, so that's its advantages. All along and you have a one-on-one interaction with the physiotherapist, dedicated time with them. So, I found lots of pluses in this online program."

P6 *"Really, it's very good to me. These are the very good exercise. It's enough for myself. I'm very active now. I like to do all these exercises. Easy to do. That's not so hard. That is very nice. I can handle this easily."*

Lack of requirements for special equipment

The exercises included in the program used easily available household objects for exercise delivery. There was no need for special equipment, which was appreciated by the participants.

P4 *"I find there is better equipment in the hospital. In home, there is only minimum equipment. If there was equipment at home, then it would be easy. There is no large difference. In particular, more equipment is used in clinics. At home, there is very little to no equipment due. Exercises are similar, I feel. I cannot make out any difference when performing exercises at home."*

Additional training resources

The participants were provided with an exercise instruction manual with illustrations and descriptions of all the included exercises with safety instructions. The participants were also provided with prerecorded videos of the exercise demonstration. These additional training resources were found to be detailed and self-explanatory. The categorization of exercises was noted to be of assistance. Some participants preferred watching the videos over reading the manual. The participants preferred a printed copy of the manual over a digital file.

P2 *"I'm quite happy with the illustrations given and the instructions given, which are quite explicit. So, I don't think that I would have any difficulty in following the regimen of exercises. The only thing that would be good is right in the beginning when it is demonstrated in person, it becomes easy to follow it once it's done. But to a large extent, even that may not be necessary if people follow the instructions on the illustrations. Because they seem very self-explanatory. I liked the categorization of the different types of exercises being strengthening, mobility and the balance and so, once you put it in separate categories, it becomes quite easy for me to follow on a regular basis. And overall, I think it, it's a good way that you put it across."*

P4 *"I would like a booklet of exercise in print to prevent distraction from the mobile (phone) while on the video call. To see the exercise since concentration is diverted on the mobile (phone). It is a problem."*

P6 stated, *"Exercise videos will be good to help in performing the exercise at home by ourselves. With exercise videos, it is easier than with the booklet. I will be able to exercise with the videos and the pictures in the booklet. I am very happy overall."*

5. Hedonic motivation

Positive interaction with the physiotherapist

The participants reported positive interaction with the physiotherapist, likening the interaction to being between a teacher and a student. They also appreciated patience, communication skills, and demonstrations provided by the therapist.

P3 stated, *"The session happens just the way it happens in a classroom, and it is easier to verify the doubts. The online session feels more like the happenings between a student and the teacher."*

P8 *"Since it is a video call, it is more comfortable for us to do at home than to come to the hospital. Commutation is a problem. That is why we like your online session. Patience. Communication. Demonstration"*

Enjoyment with TELEPORT-PD

In contrast to their expectations, the participants felt that the program was interesting and fresh due to the progression of exercise without making them too difficult. They appreciated the natural progression of exercise, which allowed them to ease in and keep up with the program.

P1 *"I expected a very dull, dry do this do that sort of thing, which I'd have to force myself into. And it wasn't like that at all. It was it was very interesting and. I mean, you kept it very fresh by introducing new things, and it was never difficult because I was sort of eased in*

slowly into the exercises. I never realized that I was improving. It just was so naturally done."

6. Price value

Economic value compared with in-person physiotherapy

Many participants reported saving money because they did not need to travel for their exercise sessions. Additionally, the relative savings in time and effort were appreciated as well.

P2 *"As I mentioned, as long as there is one contact session in the beginning. Where you can show it. I think that is all that is necessary. The rest can be easily performed via tele and I think it saves a lot. Effort, time, money. Therefore, there's only positive things to say about it. If it was something that was very difficult to comprehend, I can understand that you would need more supervision if things were much more sort of. Risky or dangerous, then I think it would need more supervision, but as it stands, I do not see any of those things at all, and I think that this system of trying to. Showing once and then following it up is a good idea. What I would suggest is maybe at some point where you want to check I should be able to do it with the video on my side for you to see what the exercises that I'm doing when I'm doing it OK. So that can be done after a few weeks or whatever."*

7. Habit

Motivation to exercise

The participant reported being motivated to exercise after participating in the program. They also describe it to be patient friendly and appreciate its structure. This enhanced motivation

for those who were not motivated at the beginning. Additionally, the participants reported that they are now more regular with exercise because of their introduction to the program.

P1 *"Oh it was a really good experience actually. I mean if I even go to the extent of saying it was life changing because it managed to motivate me, a person who had zero motivation for exercise and I didn't expect so much was possible from. You know, a tele, an online program, but I really benefited a lot from the program. So, it's been a really good experience for me. Like I mentioned, it's actually been life changing...I think actually the program that I've been exposed to has been very well structured. It's very patient friendly and as I mentioned when I began it, it even motivates people who are not motivated to start off with. So far, I think it's a very effective program and I can't really think of all too many modifications that are needed."*

P2 *"I think that I would be more. I'm more involved in the physio now than I have ever been because my diagnosis of Parkinson's was made five years ago and I was advised exercises from that point itself, but I must admit that I was never very regular because to get into a routine to do it is not easy. It's not the exercise themself being difficult, it is the motivation and the sort of getting used to the idea of doing it. But since the you know, we started with this program of the tele physio. I think I've been much more faithful about it. And so, to that extent, I think it's positive only. I certainly would like to continue it and I would certainly recommend it to others who are in the same situation and I would like some contact maybe once a month or something to know that things are going on. Yes. Yes. Any hiccups at some point of time."*

Customizability of TELEPORT-PD

Since the program was developed specifically to address the requirements of a low-resource setting, it was readily applicable to the current patient group. The exercise sequence was noted to be adequately paced. Additionally, the flexibility of the number of sessions and their timing was appreciated.

P1 *"Actually, from what I knew of, there are lots of home based and even free exercise programs in the West, particularly in America and so on. They have lots of options, lots of really good organizations with lots of really good programs. But if you if in India I just didn't find anything, anything that was doable or viable. So, I think there's a big need for the development of online exercise programs in Parkinson's because there must be lots of people like me who are looking for this option. And it's just not available at the present time, so it would be great if you could, you know, develop something that's accessible to lots of people."*

P2 *"...There's really no limitation of how many sessions I can do in the week and so on because I'm doing it at home…I think the current order is quite good. I'm happy…I think it's quite safe. I think it's adequately paced…For example, the strengthening exercise, I can do the upper and lower limbs comfortably in half an hour…That's probably a good amount of time that I can spend at doing exercises in either the morning or the evening..."*

Willingness to continue the program

The participants were willing to continue the exercise program because of its various benefits. They would also suggest that others with PD participate in the program. The participants appreciated the one-on-one sessions, which made interaction with the physiotherapist easier, leading to motivation and easier learning. Their opinion was not

positive toward group telerehabilitation sessions, especially for individuals who are new to exercising via remote guidance.

P1 *"Yeah, definitely. I will continue. And I've already given it a lot of publicity. I've talked to lots of people telling them how effective it's been for me. I would definitely recommend it to anybody who needed this program. I would have no hesitation at all...I mean any of the exercises can be done in a group, that's true, but I think particularly from where I started out as zero, it really was a great benefit to have a one-on-one interaction with you that really helped because you feel the involvement of another person and that actually motivates you so, and you know little things that are wrong or that are that need to be corrected, are pointed out to you so that on the right you know, just as a part of the process...you correct yourself. I don't know whether that much is possible. If you are in a big group. I think maybe at a later stage, when you really know what's going on, it's OK to be in a group, but maybe not in the initial stages."*

Discussion

This qualitative study aimed to explore the perspectives of persons with Parkinson's disease (PD) on the feasibility and acceptability of a low-resource tele-assisted home exercise program (TELEPORT-PD) designed to enhance balance and functional mobility. The application of the UTAUT-2 model facilitated an in-depth examination of key factors influencing the successful implementation of TELEPORT-PD in a feasibility trial.

The participants consistently expressed satisfaction with their engagement in TELEPORT-PD, which was reinforced by perceived improvements in functional mobility, flexibility, and strength. This aligns with findings from previous qualitative studies in neurological populations, where participant satisfaction was closely linked to perceived functional benefits

[12]. These insights underscore the importance of demonstrating clinical effectiveness during program development to enhance acceptance and sustained engagement. This could be achieved by objectively measuring participant satisfaction with telerehabilitation [13].

Technical challenges and comparisons with in-person physiotherapy shaped participants' expectations regarding the effort required for engagement. Reliable internet connectivity has emerged as a crucial determinant of effective program delivery, highlighting the risk posed by inadequate digital infrastructure. Additionally, limited technological proficiency was identified as a barrier to participation. Deficiencies in appropriate technology and associated factors are known limitations of deploying a telerehabilitation intervention in a low-resource setting [14]. Providing structured training and involving caregivers in setup and troubleshooting are pivotal in mitigating these challenges. At the same time, home-based participation minimized travel-related burdens, conserving time and energy that could be redirected toward exercise performance - a critical consideration for individuals with PD experiencing mobility restrictions.

The active involvement of physiotherapists and the supportive presence of caregivers played a significant role in participant engagement. Compared with unsupervised exercise, supervised exercise programs have yielded better outcomes [15]. The physiotherapists' clear instruction delivery and live demonstration of exercise fostered a strong therapeutic alliance, reinforcing motivation. Some participants noted that performing exercises alongside the physiotherapist resembled action observation therapy, potentially enhancing adherence.

While participants did not perceive the caregiver as essential for exercise performance, their role in ensuring safety and providing reassurance was recognized as beneficial.

The simplicity of TELEPORT-PD, its reliance on readily available household items, and the provision of additional training resources facilitated ease of use. Familiarity with WhatsApp Messenger as a communication platform reduced the learning curve associated with the

program. The exercises were designed to be simple and structured, with a focus on aerobic, resistance, flexibility, balance, and functional mobility training, as well as relaxation techniques. Supplementary materials, including printed and digital manuals with illustrations and prerecorded videos, empowered participants to take an active role in self-management, potentially fostering long-term adherence.

Enjoyment and positive interactions with the physiotherapist emerged as key motivators. The participants described the physiotherapist’s role as akin to that of a teacher, emphasizing patience and effective communication. Effective communication by physiotherapists has been shown to improve a person’s experience with physiotherapy [16]. Initial concerns about the program being monotonous or overly demanding were alleviated by its structured progression, which maintained engagement while ensuring a gradual increase in exercise intensity.

Economic considerations were a significant factor influencing program acceptability. The cost-effectiveness of telerehabilitation, particularly in terms of reduced travel expenses and time savings, has been well documented [4–6]. The financial and logistical advantages of TELEPORT-PD may enhance long-term adherence, particularly for individuals with PD who require sustained rehabilitation.

Motivation and program customizability are central to habit formation. The participants expressed a willingness to continue engaging in the program beyond the study period, reinforcing the potential for long-term adoption. Given the known challenges in sustaining exercise motivation among individuals with PD [17], the flexibility of TELEPORT-PD, including real-time feedback and exercise modifications on the basis of participant needs, appeared to foster a sense of empowerment. Safety considerations, such as adequate rest periods and support mechanisms, further reinforced participant confidence and program adherence.

Overall, TELEPORT-PD addresses critical rehabilitation needs in resource-constrained settings. Future research should explore additional outcomes beyond balance and functional mobility to optimize the program's impact.

Strengths and Limitations

This study provides novel insights into the perspectives of people with PD regarding a low-resource tele-rehabilitation programme, an area that has received limited attention. To the best of our knowledge, this is the first qualitative study in the region to explore this topic. The use of the UTAUT-2 model allowed for a comprehensive exploration of factors influencing program feasibility, whereas adherence to the COREQ checklist ensured rigor in data reporting.

However, certain limitations must be acknowledged. The participants' perspectives were shaped by their first exposure to telerehabilitation within a brief two-week feasibility trial, which may have influenced their overwhelmingly positive responses. However, long-term acceptability and sustained engagement remain unexplored. Additionally, all participants had internet access, smart devices, and caregiver support, which may limit the generalizability of the findings to broader PD populations lacking these resources. Furthermore, both the therapy sessions and the subsequent interviews were conducted by the same investigator, which may have introduced bias in the participants' responses. Given the feasibility constraints of the study, this limitation was unavoidable.

Recommendations for future research

Future studies should focus on identifying and addressing the specific needs of persons with PD during the early development stages of telerehabilitation programs. Further research

should evaluate long-term adherence, explore strategies to support participants with limited digital literacy or caregiver assistance, and investigate broader functional and psychosocial outcomes.

Conclusion

The participants expressed high levels of satisfaction with TELEPORT-PD, citing its accessibility, convenience, and adaptability. While initial technological barriers posed challenges, structured in-person familiarization facilitated a smooth transition to remote supervision via WhatsApp video calls. The program fostered motivation, engagement, and a sense of empowerment, with many participants expressing a desire for continued participation. These findings highlight the potential of low-resource telerehabilitation as a viable strategy for addressing rehabilitation needs in persons with PD, particularly in settings with limited access to in-person physiotherapy.

Acknowledgements

The authors thank Dr. Preetha R. and Dr. Sumita Rege for validating the interview guide for this study, as well as their suggestions for the conduct of the interviews.

References

1. Dorsey ER, Sherer T, Okun MS, Bloem BR. The Emerging Evidence of the Parkinson Pandemic. J Parkinsons Dis. 2018;8: S3–S8. doi:10.3233/JPD-181474
2. Yang Q, Chang X, Li S, Li X, Kang C, Yuan W, et al. Disease Burden of Parkinson's Disease in Asia and Its 34 Countries and Territories from 1990 to 2021: Findings from

the Global Burden of Disease Study 2021. Neuroepidemiology. 2024; 1–33. doi:10.1159/000542606

3. Özden F, Özkeskin M, Ak SM. Physical exercise intervention via telerehabilitation in patients with neurological disorders: a narrative literature review. Egypt J Neurol Psychiatr Neurosurg. 2022;58: 26. doi:10.1186/s41983-022-00461-1
4. Baffert S, Hadouiri N, Fabron C, Burgy F, Cassany A, Kemoun G. Economic Evaluation of Telerehabilitation: Systematic Literature Review of Cost-Utility Studies. JMIR Rehabil Assist Technol. 2023;10: e47172. doi:10.2196/47172
5. Grigorovich A, Xi M, Lam N, Pakosh M, Chan BCF. A systematic review of economic analyses of home-based telerehabilitation. Disabil Rehabil. 2022;44: 8188–8200. doi:10.1080/09638288.2021.2019327
6. Mousavi Baigi SF, Mousavi AS, Kimiafar K, Sarbaz M. Evaluating the cost effectiveness of tele-rehabilitation: A systematic review of randomized clinical trials. Frontiers in Health Informatics. 2022;11.
7. Hoddinott P. A new era for intervention development studies. Pilot Feasibility Stud. 2015;1: 36. doi:10.1186/s40814-015-0032-0
8. O'Cathain A, Croot L, Duncan E, Rousseau N, Sworn K, Turner KM, et al. Guidance on how to develop complex interventions to improve health and healthcare. BMJ Open. 2019;9: e029954. doi:10.1136/bmjopen-2019-029954
9. Tong A, Sainsbury P, Craig J. Consolidated criteria for reporting qualitative research (COREQ): a 32-item checklist for interviews and focus groups. International Journal for Quality in Health Care. 2007;19: 349–357. doi:10.1093/intqhc/mzm042
10. Venkatesh V, Thong JYL, Xu X. Consumer acceptance and use of information technology: extending the unified theory of acceptance and use of technology. MIS Quarterly. 2012; 157–178.

11. Ouédraogo F, Auger L-P, Moreau E, Côté O, Guerrera R, Rochette A, et al. Acceptability of Telerehabilitation: experiences and perceptions by individuals with stroke and caregivers in an early supported discharge program. Healthcare. 2024;12: 365.

12. Federico S, Cacciante L, Cieślik B, Turolla A, Agostini M, Kiper P, et al. Telerehabilitation for neurological motor impairment: a systematic review and meta-analysis on quality of life, satisfaction, and acceptance in stroke, multiple sclerosis, and Parkinson's disease. J Clin Med. 2024;13: 299.

13. Eldemir S, Eldemir K, Saygili F, Ozkul C, Kasikci M, Yilmaz R, et al. Development, reliability, and validity of the telerehabilitation satisfaction questionnaire in neurological diseases. Int J Med Inform. 2024;191: 105578. doi:10.1016/j.ijmedinf.2024.105578

14. Leochico CFD, Espiritu AI, Ignacio SD, Mojica JAP. Challenges to the Emergence of Telerehabilitation in a Developing Country: A Systematic Review. Front Neurol. 2020;11. Available: https://www.frontiersin.org/journals/neurology/articles/10.3389/fneur.2020.01007

15. Lacroix A, Kressig RW, Muehlbauer T, Gschwind YJ, Pfenninger B, Bruegger O, et al. Effects of a Supervised versus an Unsupervised Combined Balance and Strength Training Program on Balance and Muscle Power in Healthy Older Adults: A Randomized Controlled Trial. Gerontology. 2015;62: 275–288. doi:10.1159/000442087

16. Potter M, Gordon S, Hamer P. The physiotherapy experience in private practice: The patients' perspective. Australian Journal of Physiotherapy. 2003;49: 195–202. doi:10.1016/S0004-9514(14)60239-7

17. Ellis T, Boudreau JK, DeAngelis TR, Brown LE, Cavanaugh JT, Earhart GM, et al. Barriers to Exercise in People With Parkinson Disease. Phys Ther. 2013;93: 628–636. doi:10.2522/ptj.20120279

Click here to access/download
Supporting Information
S1 Interview Guide.docx

Click here to access/download
Supporting Information
S2 COREQ Checklist.docx
