# Supplementary material for: A qualitative exploration of the low-resource tele-assisted home exercise program for balance and functional mobility in Parkinson’s disease (TELEPORT-PD)
Source: PLOS Digit Health. 2026 Jun 30;5(6):e0001536. doi: 10.1371/journal.pdig.0001536 (PMC13318033; doi:10.1371/journal.pdig.0001536)
Supplement: Attachment — Submitted filename: Reviewer comments.docx [file pdig.0001536.s005.docx]

**General changes suggested by Journal**

1. **Please add a full list of legends for your Supporting Information files after the references list.**

*List of legends was added for Supporting information files after the References.*

2. **Please provide an Author Summary**

*A plain language Author Summary has been included between the Abstract and Introduction.*

**Response to Reviewer 1**

Thank you for your feedback. We are grateful and have incorporated your suggestions as below:

1. **A first critical point concerns the sample size and the limited duration of the study. Only 8 people were included, and the intervention period was two weeks. Although this may be acceptable in a preliminary qualitative study, it is necessary to emphasize more clearly how these limitations reduce the transferability and generalizability of the results. It would be appropriate to mention this limitation not only in the Discussion section, but also in the Abstract, so that the reader immediately understands the exploratory nature of the work.**

*The small sample size has been highlighted in more detail in the Discussion and Abstract with a reference to the exploratory nature of the work.*

2. **A second important element is the role of the researcher in the interviews. The same people who conducted the exercise sessions also carried out the qualitative interviews. This introduces an obvious risk of social desirability bias: participants may have reported more positive experiences to please the physiotherapist. The authors mention this possibility in the Limitations section, but the discussion appears rather brief. It would be important to expand on this point and indicate what strategies could have been used to reduce the impact of such bias (e.g., the use of independent interviewers or data triangulation).**

*The Discussion now mentions the risk of using the therapist as the interviewer. The point has been expanded on accordingly with a suggestion for reduction of bias in future work.*

3. **Methodologically, data analysis was conducted using Microsoft Excel. Although this is not incorrect in itself, it may seem unusual compared to software dedicated to qualitative analysis (such as NVivo or Atlas.ti). I would suggest better justifying this choice by explaining how the transparency of the coding process, the management of discrepancies and the traceability of decisions were ensured.**

*The use of Excel has been justified through mention of transparency in using audit log on the master spreadsheet.*

4. **Another point that deserves further consideration is the choice of the UTAUT-2 model. It is certainly interesting to apply this framework to the field of tele-rehabilitation, but the manuscript does not clearly explain why this particular model was chosen over others available for the analysis of technology acceptance. A more detailed justification would strengthen the theoretical consistency of the study and help the reader better understand the methodological choices.**

*The decision to select UTAUT-2 model has been expanded on to provide better explanation that led to its selection.*

5. **Finally, the almost exclusively positive nature of the results is striking. All participants expressed high satisfaction, with very little criticism. This may depend on contextual factors (the presence of the physiotherapist-interviewer, the small and selected sample, the brevity of the programme) and deserves more critical discussion in the Discussion section. It would be useful, for example, to point out that such positive responses may not reflect the experiences of patients with fewer technological resources, less available caregivers or less initial motivation.**

*The highly positive feedback of participants has been critically discussed in Discussion and the lack of representation for lesser motivated persons with Parkinson’s disease with poor access to technology.*

6. **From a formal point of view, the title is rather long and dense with information. A more streamlined version, which retains the acronym TELEPORT-PD but simplifies the description of the outcomes, could make it more immediate and readable.**

*The Title has been shortened as indicated*

7. **In the Abstract, the description of the results is clear, but there is no reference to the main methodological limitations (small sample size, short duration, risk of bias). A brief mention would increase the transparency of the work.**

*The Abstract has been edited to reflect the limited generalizability of the findings*

8. **The introduction is well written and documented, but could be strengthened by including not only the most recent references (2023–2024), but also more established studies on the role of tele-rehabilitation in Parkinson's disease.**

*The Introduction now mentions additional recent work done on Parkinson’s telerehabilitation.*

9. **With regard to the Methods, it would be useful to include an example of a guiding question from the interview guide, or at least refer explicitly to the supplementary material. This would make the data collection process clearer.**

*The Interview Guide has now been explicitly referred to in-text via citation.*

10. **In the Results section, the participants' quotes are very rich but in some cases are excessively long and redundant. Shortening them, while maintaining the original meaning, would improve the readability of the text.**

*The quotes have been shortened to improve readability*

11. **The Discussion is well structured, but would benefit from a more critical comparison with other similar qualitative studies, not only on Parkinson's but also on other neurological conditions. This would allow for better contextualization of the results and highlight similarities or differences with existing literature.**

*The Discussion now includes a comparative analysis of other studies in PD as well as stroke and MS.*

12. **Finally, from a linguistic point of view, the English style is generally clear and fluid, but some sentences could be made more concise to improve readability.**

*Sentences were shortened as indicated for better readability.*

**Response to Reviewer 2**

Thank you for your feedback. We appreciate the positive comments on the manuscript.

**Response to Reviewer 3**

Thank you for your feedback. We are grateful and have incorporated your suggestions as below:

1. **The introduction provides a strong rationale linking the global and regional burden of Parkinson’s disease to the need for telerehabilitation solutions. However, the section could benefit from smoother transitions and a more explicit statement of the unique contribution of the TELEPORT-PD program in the context of existing literature.**

*The Introduction has been revised as indicated for smoother transition and an explicit statement on the TELEPORT-PD program’s contribution in context of existing work.*

2. **The validation of the interview guide by two experienced qualitative researchers is appropriate, but it would be useful to specify their institutional roles and the criteria used for validation. The interview guide was pilot tested on two individuals, it should be clearly stated that the pilot data were not included in the main dataset. The translation of non-English transcripts into English by a bilingual native speaker is commendable; however, it is unclear whether back translation was performed to ensure linguistic accuracy. The transcripts were coded independently by two authors using Microsoft Excel. While this is acceptable, Excel may be considered a basic tool for qualitative coding. While the use of the UTAUT-2 model provides a strong theoretical framework, the manuscript could better explain how this model was adapted to the context of telerehabilitation acceptance among individuals with Parkinson’s disease**

*The institutional roles of the researchers involved in validation has been provided. The point of back translation was missed in the initial manuscript and has been added here. The use of Excel has been strengthened by the use of audit log function on the master spreadsheet. The choice of using UTAUT-2 model has been justified in a revised statement.*

3. **While the UTAUT-2 model was applied effectively in the analysis, the Discussion could elaborate on how each construct (e.g., performance expectancy, effort expectancy, social influence) emerged from the participants’ perspectives. Currently, the discussion is mostly thematic rather than model-driven. The Discussion tends to repeat some descriptive information that was already provided in the Results section (e.g., participants’ satisfaction, caregiver involvement). Consider summarizing these points more concisely and focusing on interpretation rather than restating results. The manuscript nicely cites previous studies on telerehabilitation and neurological rehabilitation (refs [4–6], [12–17]), but the discussion could be strengthened by contrasting TELEPORT-PD findings with telehealth studies in Parkinson’s disease specifically (e.g., motor outcomes, digital literacy barriers, long-term adherence patterns).**

*The Result and Discussion have been revised as per suggestion to improve readability and avoid repetition. More critical discussion points have been added as indicated. The findings of TELEPORT-PD have been compared to results from other studies in PD, in addition to other neurological conditions.*

4. **The strengths are well summarized, but the limitations section could also discuss the relatively short intervention duration (two weeks) as a constraint in assessing sustained engagement. Additionally, consider noting that participants’ positive feedback may have been influenced by social desirability bias. The authors acknowledge that the same investigator conducted both the intervention and interviews, which may have introduced bias. This is commendable transparency, but it would strengthen the discussion to elaborate briefly on strategies taken to minimize such bias (e.g., reflexive journaling, peer debriefing, or triangulation).**

*The Limitations are revised to highlight the shorter intervention period as a constraint. The contribution of social desirability bias making generalization difficult is now acknowledged. Furthermore, as per suggestion, strategies to minimize such bias are now mentioned.*

5. **The recommendations for future research are logical and relevant. You might expand this section by emphasizing the potential scalability of TELEPORT-PD in other low-resource regions and how co-design with patients could further enhance usability and sustainability.**

*The Recommendations for future research now emphasize the scalability potential of TELEPORT-PD and the importance of co-designing with patients and sustainability.*

6. **The conclusion effectively summarizes the main findings, but you could make it stronger by explicitly linking back to the study aim (line 461) and by stating how TELEPORT-PD contributes to bridging the gap in telerehabilitation accessibility in low-resource contexts.**

*The Conclusion has been revised to link back to the aim of the study as well as includes how the program bridges the gap in telerehabilitation accessibility in other low-resource settings.*
