# Supplementary material for: A qualitative exploration of the low-resource tele-assisted home exercise program for balance and functional mobility in Parkinson’s disease (TELEPORT-PD)
Source: PLOS Digit Health. 2026 Jun 30;5(6):e0001536. doi: 10.1371/journal.pdig.0001536 (PMC13318033; doi:10.1371/journal.pdig.0001536)
Supplement: Attachment — Submitted filename: Response to Reviewer comments - 29-4-26.docx [file pdig.0001536.s006.docx]

**Response to Reviewer 4**

Thank you for your feedback. We are grateful and have incorporated your suggestions as below:

- The discussion now includes deeper critical reflection on social desirability bias, novelty effects, and contextual influences.
- We have included mention of the researcher positionality leading to potential bias and listed methods used to address them. The influence of caregiver presence has been added.
- The specific constructs and behavioural intention stemming from UTAUT-2 have been discussed.
- The limited applicability of this study to broader low-resource settings has been acknowledged.
- The full de-identified transcripts will be shared by the corresponding author on request.

**Response to Reviewer 5**

Thank you for your feedback. We are grateful and have incorporated your suggestions as below:

- The abstract now includes the suggested statement.
- The quotations have been edited for concision to enhance readability.
- Language editing has been done.
