# Supplementary material for: A qualitative exploration of the low-resource tele-assisted home exercise program for balance and functional mobility in Parkinson’s disease (TELEPORT-PD)
Source: PLOS Digit Health. 2026 Jun 30;5(6):e0001536. doi: 10.1371/journal.pdig.0001536 (PMC13318033; doi:10.1371/journal.pdig.0001536)
Supplement: Peer review history

# Peer review: Decision Letter 0

**DOI:** 10.1371/journal.pdig.0001536.r001

**Authors:** Zhao Ni, Raquel Simões de Almeida

23 Nov 2025

PDIG-D-25-00578

Exploring participants’ perspectives toward a low-resource tele-assisted home exercise program for balance and functional mobility in Parkinson’s disease (TELEPORT-PD): a qualitative study from India

PLOS Digital Health

Dear Dr. Natarajan,

Thank you for submitting your manuscript to PLOS Digital Health. After careful consideration, we feel that it has merit but does not fully meet PLOS Digital Health's publication criteria as it currently stands. Therefore, we invite you to submit a revised version of the manuscript that addresses the points raised during the review process.

Please submit your revised manuscript within 60 days Jan 22 2026 11:59PM. If you will need more time than this to complete your revisions, please reply to this message or contact the journal office at digitalhealth@plos.org. When you're ready to submit your revision, log on to https://www.editorialmanager.com/pdig/ and select the 'Submissions Needing Revision' folder to locate your manuscript file.

Please include the following items when submitting your revised manuscript:

* A rebuttal letter that responds to each point raised by the editor and reviewer(s). You should upload this letter as a separate file labeled 'Response to Reviewers'. This file does not need to include responses to any formatting updates and technical items listed in the 'Journal Requirements' section below.

* A marked-up copy of your manuscript that highlights changes made to the original version. You should upload this as a separate file labeled 'Revised Manuscript with Track Changes'.

* An unmarked version of your revised paper without tracked changes. You should upload this as a separate file labeled 'Manuscript'.

If you would like to make changes to your financial disclosure, competing interests statement, or data availability statement, please make these updates within the submission form at the time of resubmission. Guidelines for resubmitting your figure files are available below the reviewer comments at the end of this letter.

We look forward to receiving your revised manuscript.

Kind regards,

Raquel Simões de Almeida, PhD

Academic Editor

PLOS Digital Health

Raquel Simões de Almeida

Academic Editor

PLOS Digital Health

Leo Anthony Celi

Editor-in-Chief

PLOS Digital Health

orcid.org/0000-0001-6712-6626

**Journal Requirements:**

1. We have noticed that you have uploaded Supporting Information files, but you have not included a list of legends. Please add a full list of legends for your Supporting Information files after the references list.

2. Please provide an Author Summary. This should appear in your manuscript between the Abstract (if applicable) and the Introduction, and should be 150–200 words long. The aim should be to make your findings accessible to a wide audience that includes both scientists and non-scientists. Sample summaries can be found on our website under Submission Guidelines:

https://journals.plos.org/digitalhealth/s/submission-guidelines#loc-parts-of-a-submission

3. If the reviewer comments include a recommendation to cite specific previously published works, please review and evaluate these publications to determine whether they are relevant and should be cited. There is no requirement to cite these works unless the editor has indicated otherwise.

**Additional Editor Comments:**

In addition to the reviewers' comments, it is important, particularly as this is a qualitative study, to explore the issues surrounding researcher reflexivity.

[Note: HTML markup is below. Please do not edit.]

**Reviewers' Comments:**

Reviewer's Responses to Questions

**Comments to the Author**

1. Does this manuscript meet PLOS Digital Health’s publication criteria? Is the manuscript technically sound, and do the data support the conclusions? The manuscript must describe methodologically and ethically rigorous research with conclusions that are appropriately drawn based on the data presented.

Reviewer #1: Partly

Reviewer #2: Yes

Reviewer #3: Yes

**********

2. Has the statistical analysis been performed appropriately and rigorously?

Reviewer #1: Yes

Reviewer #2: Yes

Reviewer #3: Yes

**********

3. Have the authors made all data underlying the findings in their manuscript fully available (please refer to the Data Availability Statement at the start of the manuscript PDF file)?

The PLOS Data policy requires authors to make all data underlying the findings described in their manuscript fully available without restriction, with rare exception. The data should be provided as part of the manuscript or its supporting information, or deposited to a public repository. For example, in addition to summary statistics, the data points behind means, medians and variance measures should be available. If there are restrictions on publicly sharing data—e.g. participant privacy or use of data from a third party—those must be specified.

Reviewer #1: Yes

Reviewer #2: Yes

Reviewer #3: Yes

**********

4. Is the manuscript presented in an intelligible fashion and written in standard English?

PLOS Digital Health does not copyedit accepted manuscripts, so the language in submitted articles must be clear, correct, and unambiguous. Any typographical or grammatical errors should be corrected at revision, so please note any specific errors here.

Reviewer #1: Yes

Reviewer #2: Yes

Reviewer #3: Yes

**********

5. Review Comments to the Author

Please use the space provided to explain your answers to the questions above. You may also include additional comments for the author, including concerns about dual publication, research ethics, or publication ethics. (Please upload your review as an attachment if it exceeds 20,000 characters)

Reviewer #1: This manuscript addresses an important and timely topic, namely the feasibility and acceptability of a low-resource tele-assisted home exercise program for individuals with Parkinson’s disease in India. The study is well written, methodologically sound overall, and adheres to COREQ guidelines. The use of the UTAUT-2 framework is original and contributes to the novelty of the work.

However, there are notable limitations. The sample size is very small (n=8) and limited to a two-week feasibility trial, which raises questions about the generalizability of the findings. Moreover, the dual role of the main researcher as both interventionist and interviewer introduces a risk of bias that needs to be more thoroughly discussed. While these constraints may be acceptable for an exploratory qualitative study, they should be highlighted more clearly in the Abstract and Discussion.

The results are strikingly positive, with minimal critical feedback from participants. This may reflect selection or interview bias and warrants deeper reflection. Additionally, the rationale for using Microsoft Excel for qualitative analysis and the choice of UTAUT-2 over other theoretical frameworks should be better justified.

Overall, I believe this manuscript provides valuable insights into tele-rehabilitation in low-resource settings and could be a good contribution to the field once the issues above are addressed.

Major Revision

1. A first critical point concerns the sample size and the limited duration of the study. Only 8 people were included, and the intervention period was two weeks. Although this may be acceptable in a preliminary qualitative study, it is necessary to emphasize more clearly how these limitations reduce the transferability and generalizability of the results. It would be appropriate to mention this limitation not only in the Discussion section, but also in the Abstract, so that the reader immediately understands the exploratory nature of the work.

2. A second important element is the role of the researcher in the interviews. The same people who conducted the exercise sessions also carried out the qualitative interviews. This introduces an obvious risk of social desirability bias: participants may have reported more positive experiences to please the physiotherapist. The authors mention this possibility in the Limitations section, but the discussion appears rather brief. It would be important to expand on this point and indicate what strategies could have been used to reduce the impact of such bias (e.g., the use of independent interviewers or data triangulation).

3. Methodologically, data analysis was conducted using Microsoft Excel. Although this is not incorrect in itself, it may seem unusual compared to software dedicated to qualitative analysis (such as NVivo or Atlas.ti). I would suggest better justifying this choice by explaining how the transparency of the coding process, the management of discrepancies and the traceability of decisions were ensured.

4. Another point that deserves further consideration is the choice of the UTAUT-2 model. It is certainly interesting to apply this framework to the field of tele-rehabilitation, but the manuscript does not clearly explain why this particular model was chosen over others available for the analysis of technology acceptance. A more detailed justification would strengthen the theoretical consistency of the study and help the reader better understand the methodological choices.

5. Finally, the almost exclusively positive nature of the results is striking. All participants expressed high satisfaction, with very little criticism. This may depend on contextual factors (the presence of the physiotherapist-interviewer, the small and selected sample, the brevity of the programme) and deserves more critical discussion in the Discussion section. It would be useful, for example, to point out that such positive responses may not reflect the experiences of patients with fewer technological resources, less available caregivers or less initial motivation.

Minor Revision

1. From a formal point of view, the title is rather long and dense with information. A more streamlined version, which retains the acronym TELEPORT-PD but simplifies the description of the outcomes, could make it more immediate and readable.

2. In the Abstract, the description of the results is clear, but there is no reference to the main methodological limitations (small sample size, short duration, risk of bias). A brief mention would increase the transparency of the work.

3. The introduction is well written and documented, but could be strengthened by including not only the most recent references (2023–2024), but also more established studies on the role of tele-rehabilitation in Parkinson's disease.

4. With regard to the Methods, it would be useful to include an example of a guiding question from the interview guide, or at least refer explicitly to the supplementary material. This would make the data collection process clearer.

5. In the Results section, the participants' quotes are very rich but in some cases are excessively long and redundant. Shortening them, while maintaining the original meaning, would improve the readability of the text.

6. The Discussion is well structured, but would benefit from a more critical comparison with other similar qualitative studies, not only on Parkinson's but also on other neurological conditions. This would allow for better contextualization of the results and highlight similarities or differences with existing literature.

7. Finally, from a linguistic point of view, the English style is generally clear and fluid, but some sentences could be made more concise to improve readability.

Reviewer #2: It is a well executed qulitative study of a digital health tool for Parkinson's Disease. The results of the paper show that low-resource telerehabilitation can be perceived as effective in relation to the rehabilitation needs of persons with PD. Apart from it's usefull claims in this area of research it is also an original attempt because it uses qualitative methods in it's approach. Although previous literature has been taken into account to a significant degree it may have been usefull to extend the discussion section a bit more by further relating the findings with previous research.

As a qualitative study it sets the basis for further research into the effectiveness of this particular intervention. The coclusions that were drawn in the study were sufficiently supported by the data analyses. There are certain limitations in the design of the study. Such as the fact that the same person conducted the therapy sessions and the interviews, which may have introduced bias in the participants responces. As well as the fact that the participants had already established a significant level of digital readiness before the intervention was implemented, which limits the generalisability of the findings. However, both limitations were recognised and taken into account by the researchers.

The research protocols of the qualitative study where thoroughly explained and followed. In my opinion, the methodology is sufficiently described for the experiment to be reproduced. Both the organisation of the manuscript and the language used allow non-specialists to benefit from it. Overall, it is a sound research attempt with usefull findings.

Reviewer #3: Introduction:

-The introduction provides a strong rationale linking the global and regional burden of Parkinson’s disease to the need for telerehabilitation solutions. However, the section could benefit from smoother transitions and a more explicit statement of the unique contribution of the TELEPORT-PD program in the context of existing literature.

Methods:

-The validation of the interview guide by two experienced qualitative researchers is appropriate, but it would be useful to specify their institutional roles and the criteria used for validation.

-The interview guide was pilot tested on two individuals, it should be clearly stated that the pilot data were not included in the main dataset.

-The translation of non-English transcripts into English by a bilingual native speaker is commendable; however, it is unclear whether back translation was performed to ensure linguistic accuracy.

-The transcripts were coded independently by two authors using Microsoft Excel. While this is acceptable, Excel may be considered a basic tool for qualitative coding.

-While the use of the UTAUT-2 model provides a strong theoretical framework, the manuscript could better explain how this model was adapted to the context of telerehabilitation acceptance among individuals with Parkinson’s disease

Results:

-The study included only eight participants, which may raise concerns about whether data saturation was achieved.

Düscussion:

-While the UTAUT-2 model was applied effectively in the analysis, the Discussion could elaborate on how each construct (e.g., performance expectancy, effort expectancy, social influence) emerged from the participants’ perspectives. Currently, the discussion is mostly thematic rather than model-driven.

-The Discussion tends to repeat some descriptive information that was already provided in the Results section (e.g., participants’ satisfaction, caregiver involvement). Consider summarizing these points more concisely and focusing on interpretation rather than restating results.

-The manuscript nicely cites previous studies on telerehabilitation and neurological rehabilitation (refs [4–6], [12–17]), but the discussion could be strengthened by contrasting TELEPORT-PD findings with telehealth studies in Parkinson’s disease specifically (e.g., motor outcomes, digital literacy barriers, long-term adherence patterns).

Strengths and Limitations:

-The strengths are well summarized, but the limitations section could also discuss the relatively short intervention duration (two weeks) as a constraint in assessing sustained engagement. Additionally, consider noting that participants’ positive feedback may have been influenced by social desirability bias.

-The authors acknowledge that the same investigator conducted both the intervention and interviews, which may have introduced bias. This is commendable transparency, but it would strengthen the discussion to elaborate briefly on strategies taken to minimize such bias (e.g., reflexive journaling, peer debriefing, or triangulation).

Recommendations:

-The recommendations for future research are logical and relevant. You might expand this section by emphasizing the potential scalability of TELEPORT-PD in other low-resource regions and how co-design with patients could further enhance usability and sustainability.

Conclusion:

-The conclusion effectively summarizes the main findings, but you could make it stronger by explicitly linking back to the study aim (line 461) and by stating how TELEPORT-PD contributes to bridging the gap in telerehabilitation accessibility in low-resource contexts.

**********

6. PLOS authors have the option to publish the peer review history of their article (what does this mean?). If published, this will include your full peer review and any attached files.

**Do you want your identity to be public for this peer review?**  If you choose “no”, your identity will remain anonymous but your review may still be made public.

For information about this choice, including consent withdrawal, please see our Privacy Policy.

Reviewer #1: No

Reviewer #2: No

Reviewer #3: **Yes:**  Kubra Kardeş

**********

[NOTE: If reviewer comments were submitted as an attachment file, they will be attached to this email and accessible via the submission site. Please log into your account, locate the manuscript record, and check for the action link "View Attachments". If this link does not appear, there are no attachment files.]

**Figure resubmission:**

While revising your submission, we strongly recommend that you use PLOS’s NAAS tool (https://ngplosjournals.pagemajik.ai/artanalysis) to test your figure files. NAAS can convert your figure files to the TIFF file type and meet basic requirements (such as print size, resolution), or provide you with a report on issues that do not meet our requirements and that NAAS cannot fix.

After uploading your figures to PLOS’s NAAS tool - https://ngplosjournals.pagemajik.ai/artanalysis, NAAS will process the files provided and display the results in the "Uploaded Files" section of the page as the processing is complete. If the uploaded figures meet our requirements (or NAAS is able to fix the files to meet our requirements), the figure will be marked as "fixed" above. If NAAS is unable to fix the files, a red "failed" label will appear above. When NAAS has confirmed that the figure files meet our requirements, please download the file via the download option, and include these NAAS processed figure files when submitting your revised manuscript.

**Reproducibility:**

To enhance the reproducibility of your results, we recommend that authors of applicable studies deposit laboratory protocols in protocols.io, where a protocol can be assigned its own identifier (DOI) such that it can be cited independently in the future. Additionally, PLOS ONE offers an option to publish peer-reviewed clinical study protocols. Read more information on sharing protocols at https://plos.org/protocols?utm_medium=editorial-email&utm_source=authorletters&utm_campaign=protocols

Attachment Submitted filename: PDIG-D-25-00578.pdf

# Author comment: Author response to Decision Letter 1

**DOI:** 10.1371/journal.pdig.0001536.r002

21 Jan 2026

Attachment Submitted filename: Reviewer comments.docx

# Peer review: Decision Letter 1

**DOI:** 10.1371/journal.pdig.0001536.r003

**Authors:** Zhao Ni, Raquel Simões de Almeida, Zhao Ni, Raquel Simões de Almeida

30 Mar 2026

PDIG-D-25-00578R1

A qualitative exploration of the low-resource tele-assisted home exercise program for balance and functional mobility in Parkinson’s disease (TELEPORT-PD)

PLOS Digital Health

Dear Dr. Natarajan,

Thank you for submitting your manuscript to PLOS Digital Health. After careful consideration, we feel that it has merit but does not fully meet PLOS Digital Health's publication criteria as it currently stands. Therefore, we invite you to submit a revised version of the manuscript that addresses the points raised during the review process.

Please submit your revised manuscript by Apr 29 2026 11:59PM. If you will need more time than this to complete your revisions, please reply to this message or contact the journal office at digitalhealth@plos.org. When you're ready to submit your revision, log on to https://www.editorialmanager.com/pdig/ and select the 'Submissions Needing Revision' folder to locate your manuscript file.

Please include the following items when submitting your revised manuscript:

* A letter that responds to each point raised by the editor and reviewer(s). You should upload this letter as a separate file labeled 'Response to Reviewers'. This file does not need to include responses to any formatting updates and technical items listed in the 'Journal Requirements' section below.

* A marked-up copy of your manuscript that highlights changes made to the original version. You should upload this as a separate file labeled 'Revised Manuscript with Track Changes'.

* An unmarked version of your revised paper without tracked changes. You should upload this as a separate file labeled 'Manuscript'.

If you would like to make changes to your financial disclosure, competing interests statement, or data availability statement, please make these updates within the submission form at the time of resubmission. Guidelines for resubmitting your figure files are available below the reviewer comments at the end of this letter.

We look forward to receiving your revised manuscript.

Kind regards,

Raquel Simões de Almeida, PhD

Academic Editor

PLOS Digital Health

Leo Anthony Celi

Editor-in-Chief

PLOS Digital Health

orcid.org/0000-0001-6712-6626

**Journal Requirements:**

If the reviewer comments include a recommendation to cite specific previously published works, please review and evaluate these publications to determine whether they are relevant and should be cited. There is no requirement to cite these works unless the editor has indicated otherwise.

**Additional Editor Comments (if provided):**

In this second round of reviews, we have received varying feedback from the reviewers; however, several key areas require further strengthening. While the study is well-grounded in the UTAUT-2 framework, the discussion needs a deeper critical reflection on potential social desirability bias and the 'novelty effect' of the intervention. It is also essential to expand on researcher reflexivity, specifically addressing how the dual role of the investigator and the presence of caregivers during interviews may have influenced the results. Furthermore, the findings should be more explicitly linked back to UTAUT-2 theoretical constructs rather than remaining largely descriptive. Finally, the authors must more clearly acknowledge the limits of transferability, as the high-functioning nature of the sample and their access to support may not reflect the broader low-resource populations.

[Note: HTML markup is below. Please do not edit.]

**Reviewers' Comments:**

Reviewer's Responses to Questions

**Comments to the Author**

1. If the authors have adequately addressed your comments raised in a previous round of review and you feel that this manuscript is now acceptable for publication, you may indicate that here to bypass the “Comments to the Author” section, enter your conflict of interest statement in the “Confidential to Editor” section, and submit your "Accept" recommendation.

Reviewer #4: (No Response)

Reviewer #5: (No Response)

Reviewer #6: All comments have been addressed

**********

2. Does this manuscript meet PLOS Digital Health’s publication criteria? Is the manuscript technically sound, and do the data support the conclusions? The manuscript must describe methodologically and ethically rigorous research with conclusions that are appropriately drawn based on the data presented.

Reviewer #4: Partly

Reviewer #5: Yes

Reviewer #6: Yes

**********

3. Has the statistical analysis been performed appropriately and rigorously?

Reviewer #4: N/A

Reviewer #5: N/A

Reviewer #6: Yes

**********

4. Have the authors made all data underlying the findings in their manuscript fully available (please refer to the Data Availability Statement at the start of the manuscript PDF file)?

The PLOS Data policy requires authors to make all data underlying the findings described in their manuscript fully available without restriction, with rare exception. The data should be provided as part of the manuscript or its supporting information, or deposited to a public repository. For example, in addition to summary statistics, the data points behind means, medians and variance measures should be available. If there are restrictions on publicly sharing data—e.g. participant privacy or use of data from a third party—those must be specified.

Reviewer #4: No

Reviewer #5: Yes

Reviewer #6: Yes

**********

5. Is the manuscript presented in an intelligible fashion and written in standard English?

PLOS Digital Health does not copyedit accepted manuscripts, so the language in submitted articles must be clear, correct, and unambiguous. Any typographical or grammatical errors should be corrected at revision, so please note any specific errors here.

Reviewer #4: Yes

Reviewer #5: Yes

Reviewer #6: No

**********

6. Review Comments to the Author

Please use the space provided to explain your answers to the questions above. You may also include additional comments for the author, including concerns about dual publication, research ethics, or publication ethics. (Please upload your review as an attachment if it exceeds 20,000 characters)

Reviewer #4: Thank you for submitting this important and timely study on TELEPORT-PD. The manuscript is well organized, clearly written, and grounded in an appropriate theoretical framework (UTAUT-2). The intervention is well described, and the qualitative methodology is transparently reported.

That said, several areas would benefit from clarification and strengthening:

Depth of analysis – The findings are positive. While acknowledged as a limitation, the discussion would benefit from deeper critical reflection on possible social desirability bias, novelty effects, and contextual influences.

Reflexivity – Since the same investigator delivered the intervention and conducted the interviews, please expand the discussion of researcher positionality and how potential bias was addressed. Clarify how caregiver presence during interviews may have influenced responses.

Theoretical integration – The UTAUT-2 framework is appropriate, but the discussion could more explicitly link findings back to specific constructs and behavioral intention rather than remaining largely descriptive.

Transferability – The sample consisted of relatively high-functioning participants with internet access and caregiver support. Please further acknowledge how this may limit applicability to broader low-resource populations.

Data availability – The Data Availability Statement indicates that all data are included, but full de-identified transcripts are not provided. Please clarify whether data sharing is restricted or consider depositing transcripts in accordance with PLOS policy.

Reviewer #5: Thank you for the opportunity to review this revised manuscript.

In my view, the authors have adequately addressed the main concerns, and the manuscript has improved in overall clarity and quality. I have two minor suggestions that may further improve the paper.

1. Abstract transparency

The abstract would be strengthened by briefly stating the main methodological limitations, as the term “exploratory” alone does not make these sufficiently clear. For example, a brief statement such as “Findings should be interpreted cautiously du to the small sample and potential response bias” would improve transparency.

2. Readability of quotations in the Results section

The Results section contains valuable participant quotations, but a few passages still contain excess detail and repeated content. Further editing for concision would enhance readability without compromising meaning.

Reviewer #6: Authors have addressed all the comments, but I still feel language editing is required for better expression of the research work done in the study.

**********

7. PLOS authors have the option to publish the peer review history of their article (what does this mean?). If published, this will include your full peer review and any attached files.

**Do you want your identity to be public for this peer review?**  If you choose “no”, your identity will remain anonymous but your review may still be made public.

For information about this choice, including consent withdrawal, please see our Privacy Policy.

Reviewer #4: No

Reviewer #5: No

Reviewer #6: **Yes:**  Insha Zahoor

**********

[NOTE: If reviewer comments were submitted as an attachment file, they will be attached to this email and accessible via the submission site. Please log into your account, locate the manuscript record, and check for the action link "View Attachments". If this link does not appear, there are no attachment files.]

**Figure resubmission:**

While revising your submission, we strongly recommend that you use PLOS’s NAAS tool (https://ngplosjournals.pagemajik.ai/artanalysis) to test your figure files. NAAS can convert your figure files to the TIFF file type and meet basic requirements (such as print size, resolution), or provide you with a report on issues that do not meet our requirements and that NAAS cannot fix.

After uploading your figures to PLOS’s NAAS tool - https://ngplosjournals.pagemajik.ai/artanalysis, NAAS will process the files provided and display the results in the "Uploaded Files" section of the page as the processing is complete. If the uploaded figures meet our requirements (or NAAS is able to fix the files to meet our requirements), the figure will be marked as "fixed" above. If NAAS is unable to fix the files, a red "failed" label will appear above. When NAAS has confirmed that the figure files meet our requirements, please download the file via the download option, and include these NAAS processed figure files when submitting your revised manuscript.

**Reproducibility:**

To enhance the reproducibility of your results, we recommend that authors of applicable studies deposit laboratory protocols in protocols.io, where a protocol can be assigned its own identifier (DOI) such that it can be cited independently in the future. Additionally, PLOS ONE offers an option to publish peer-reviewed clinical study protocols. Read more information on sharing protocols at https://plos.org/protocols?utm_medium=editorial-email&utm_source=authorletters&utm_campaign=protocols

# Author comment: Author response to Decision Letter 2

**DOI:** 10.1371/journal.pdig.0001536.r004

28 Apr 2026

Attachment Submitted filename: Response to Reviewer comments - 29-4-26.docx

# Editor report: Decision Letter 2

**DOI:** 10.1371/journal.pdig.0001536.r005

**Authors:** Zhao Ni, Raquel Simões de Almeida, Zhao Ni, Raquel Simões de Almeida, Zhao Ni, Raquel Simões de Almeida

15 Jun 2026

A qualitative exploration of the low-resource tele-assisted home exercise program for balance and functional mobility in Parkinson’s disease (TELEPORT-PD)

PDIG-D-25-00578R2

Dear Dr Natarajan,

We are pleased to inform you that your manuscript 'A qualitative exploration of the low-resource tele-assisted home exercise program for balance and functional mobility in Parkinson’s disease (TELEPORT-PD)' has been provisionally accepted for publication in PLOS Digital Health.

Before your manuscript can be formally accepted you will need to complete some formatting changes, which you will receive in a follow-up email from a member of our team.

Please note that your manuscript will not be scheduled for publication until you have made the required changes, so a swift response is appreciated.

IMPORTANT: The editorial review process is now complete. PLOS will only permit corrections to spelling, formatting or significant scientific errors from this point onwards. Requests for major changes, or any which affect the scientific understanding of your work, will cause delays to the publication date of your manuscript.

If your institution or institutions have a press office, please notify them about your upcoming paper to help maximize its impact. If they'll be preparing press materials, please inform our press team as soon as possible -- no later than 48 hours after receiving the formal acceptance. Your manuscript will remain under strict press embargo until 2 pm Eastern Time on the date of publication. For more information, please contact digitalhealth@plos.org.

Thank you again for supporting Open Access publishing; we are looking forward to publishing your work in PLOS Digital Health.

Best regards,

Raquel Simões de Almeida, PhD

Academic Editor

PLOS Digital Health

***********************************************************

**Additional Editor Comments (if provided):**

The introduction should be strengthened by clarifying why findings from studies of telerehabilitation for Parkinson's disease (PD) conducted in high-income settings may not be directly applicable to persons with PD in lower-income or resource-constrained settings.

**Reviewer Comments (if any, and for reference):**
